# A crowdsourced global data set for validating built-up surface layers

**DOI:** 10.1038/s41597-021-01105-4

**Published:** 2022-01-20

**Authors:** Linda See, Ivelina Georgieva, Martina Duerauer, Thomas Kemper, Christina Corbane, Luca Maffenini, Javier Gallego, Martino Pesaresi, Flavius Sirbu, Rekib Ahmed, Kateryna Blyshchyk, Brigitte Magori, Volodymyr Blyshchyk, Oleksandr Melnyk, Roman Zadorozhniuk, Marian-Traian Mandici, Yuan-Fong Su, Ahmed Harb Rabia, Ana Pérez-Hoyos, Roman Vasylyshyn, Chandra Kant Pawe, Svitlana Bilous, Serhii B. Kovalevskyi, Sergii S. Kovalevskyi, Kusumbor Bordoloi, Andrii Bilous, Kripal Panging, Valentyn Bilous, Reinhard Prestele, Dhrubajyoti Sahariah, Anjan Deka, Nityaranjan Nath, Rui Neves, Viktor Myroniuk, Mathias Karner, Steffen Fritz

**Affiliations:** 1 Ecosystem Services and Management Program, International Institute for Applied Systems Analysis (IIASA), Schlossplatz 1, Laxenburg, Austria; 2 European Commission, Joint Research Center, Via Enrico Fermi, 2749, I-21027 Ispra, Italy; 3 West University of Timisoara, Bulevardul Vasile Parvan no 4, Timisoara, 300323 Romania; 4 Department of Geography, Gauhati University, Jalukbari, Guwahati, Assam 781014 India; 5 Faculty of Humanities and Pedagogy, National University of Life and Environmental Sciences of Ukraine (NULESU), Heroiv Oborony 15, Kyiv, 03041 Ukraine; 6 Institute of Forestry and Landscape-Park Management, National University of Life and Environmental Sciences of Ukraine (NULESU), Heroiv Oborony 15, Kyiv, 03041 Ukraine; 7 Regional Meteorological Center Banat-Crisana, Gheorghe Adam no 15, Timisoara, 300310 Romania; 8 Department of Harbor and River Engineering, National Taiwan Ocean University, No.2 Pei-Ning Road, Keelung, 20224 Taiwan ROC; 9 National Science and Technology Center for Disaster Reduction, 9 F., No.200, Sec. 3, Beisin Rd., Xindian District, New Taipei City, 23143 Taiwan ROC; 10 Damanhour University, Faculty of Agriculture, Natural Resources & Agricultural Engineering Department, El-abaadya Campus, Damanhour, 22516 El-Behera Egypt; 11 Department of Geography, Pragjyotish College, Guwahati-09, Guwahati, Assam India; 12 Institute of Meteorology and Climate Research - Atmospheric Environmental Research (IMK-IFU), Karlsruhe Institute of Technology (KIT), Kreuzeckbahnstraße 19, 82467 Garmisch-Partenkirchen, Germany; 13 Risk and Safety Department, Higher Institute of Information and Administration Sciences, Santa Joana, 3810-488 Aveiro, Portugal; 14 Institute for Evolutionary Ecology, National Academy of Science of Ukraine, acad, Lebedeva, 37, Kyiv, 03143 Ukraine

**Keywords:** Geography, Environmental sciences

## Abstract

Several global high-resolution built-up surface products have emerged over the last five years, taking full advantage of open sources of satellite data such as Landsat and Sentinel. However, these data sets require validation that is independent of the producers of these products. To fill this gap, we designed a validation sample set of 50 K locations using a stratified sampling approach independent of any existing global built-up surface products. We launched a crowdsourcing campaign using Geo-Wiki (https://www.geo-wiki.org/) to visually interpret this sample set for built-up surfaces using very high-resolution satellite images as a source of reference data for labelling the samples, with a minimum of five validations per sample location. Data were collected for 10 m sub-pixels in an 80 × 80 m grid to allow for geo-registration errors as well as the application of different validation modes including exact pixel matching to majority or percentage agreement. The data set presented in this paper is suitable for the validation and inter-comparison of multiple products of built-up areas.

## Abstract (summary)

**Table:** 

Measurement(s)	built-up areas
Technology Type(s)	visual interpretation of satellite imagery
Factor Type(s)	geographic location
Sample Characteristic - Environment	land • area of developed space
Sample Characteristic - Location	global

Machine-accessible metadata file describing the reported data: 10.6084/m9.figshare.17068160

## Background & Summary

At present, around 55% of the world’s population lives in cities, which is predicted to increase to 68% by 2050^1^. Cities are currently responsible for between 71–76% of global CO_2_ emissions and they consume 67–76% of the world’s energy despite taking up only a small share of the Earth’s land surface^2^. With the effects of climate change (i.e., urban heat islands, sea level rises and increases in the frequency and magnitude of extreme events), cities are even more vulnerable as many are located on the coast or on the floodplains of major rivers^3,4^. Mapping the location of urban areas is increasingly important for high resolution climate modelling^5^, for climate change mitigation and adaptation strategies^6^, for the assessment of flood risk^7^, and urban and regional planning more generally^8^, among many other applications.

There have been numerous attempts in the past to map urban areas worldwide and regionally, from the artificial or built-up class in global land cover maps using remote sensing^9^ to using population data from censuses in combination with redistribution approaches^10–12^. More detailed urban mapping of urban structural types using remote sensing has also been undertaken for numerous individual cities^13^ or through initiatives such as the Urban Atlas, which has mapped all cities across the European Union (EU) with a population greater than 50K^14^, or the World Urban Database and Access Portal Tools (WUDAPT) project, which aims to map every city in the world using 10 detailed urban classes, mainly to improve urban climate modelling^5^.

More recently, with the availability of openly available high-resolution satellite imagery such as Landsat and Sentinel, and global radar data sets to identify object heights and volumes, new layers have emerged that specifically characterize the built-up surface. The Joint Research Center (JRC) of the European Commission, as part of the Group on Earth Observation’s (GEO) Human Planet Initiative, initially produced a series of built-up area grids for 1975, 1990, 2000 and 2014 available at a 30 m resolution based on multi-temporal Landsat imagery (R2018A)^15,16^. This was followed by built-up area grids derived from Sentinel-1 for 2016 (R2018A)^17,18^ and from Sentinel-2 for 2018^19^, as part of the Global Human Settlement Layer (GHSL) data package^20^. Around the same time, the German Aerospace Center (DLR) released a product called the Global Urban Footprint (GUF_DLR_v01) for the year 2011 at resolutions of 12 and 84 m^21,22^, followed by the more recent World Settlement Footprint for 2015 (WSF2015) at 10 m resolution^23^.

Although these products have been validated in different ways (see Supplementary Information (SI) for further details), the disadvantages of the approaches taken are three-fold. First, the validation data set produced for the WSF2015 was based on a stratified random sample using the product as an input, so it is not suitable for the validation of other built-up surface products. Moreover, it is not openly available for use in other validation exercises. Secondly, using the older GUF_DLR_v01 product as a reference data set for validation means there will be uncertainty due to omission and commission errors in this product as well as the temporal difference. Finally, the current validation of these products is not independent of either the product or the producer. Hence, there is a need to generate an independent, multi-purpose validation data set for assessing the quality of different data sets of built-up areas, but it will also allow for the continuous validation of future grids, e.g., to validate the annual land cover products that will be produced by the Copernicus global land service in the future that include a built-up component.

Here we present a crowdsourced, global data set that can be used to validate any remotely sensed product on built-up surfaces. A stratified-random sampling design was implemented using strata independent of current built-up surface products to produce a data set of 50 K locations globally. Using very high-resolution satellite imagery from Google Maps and Microsoft Bing Maps in Geo-Wiki^24^, the locations were visually interpreted for the presence of built-up surfaces. Change in built-up observed between pairs of images (from Google Maps and Microsoft Bing) was also recorded along with the dates of the imagery.

## Methods

### Definition of built-up areas

A built-up area is defined as an area containing any building with a roof^25^, where a building is defined as an “enclosed construction above ground, which is intended or used for the shelter of humans, animals, things or for the production of economic goods and that refer to any structure constructed or erected on its site”^26^. Note that the definition of built-up areas employed here does not include any reference to permanency so temporary structures are also included in this definition. As buildings are easily visible from very high-resolution satellite and aerial imagery, they can be visually interpreted using crowdsourcing.

### Sampling design

To produce a validation sample that is independent of the product being validated, an approach such as that implemented by Olofsson *et al*.^27^ is required, i.e., the generation of a stratified random sample based on external strata. This involves the division of the population (i.e., the collection of all pixels contained in the map) into mutually exclusive subsets (i.e., strata) within which random samples are then selected. A mask of land areas was first used to ensure that samples fell only within land-based areas. The sampling schema included a three-level stratification as follows:

- In Strata 1: we consider inhabited and non-inhabited areas as defined by the Generalized Settlement Area^28^. The latter has been generated from the union of all available data sets describing human settlements at a global scale on a grid of 1 km^2^. An inhabited cell is where at least one data set reports the presence of human settlements, whereas a non-inhabited cell is where none of the global data sets report human settlements.
- In Strata 2: we consider low, middle and high income countries on the basis of the United Nations (UN) country classification of 2015^29^.
- In Strata 3: we consider the following aggregated land cover classes as defined according to the ESA Climate Change Initiative Land Cover for 2014 at 300 m resolution^30^: class 1 includes “tree cover”; class 2 includes “grassland” and “shrubland”, class 3 includes “cropland”, class 4 includes “urban areas”, “bare areas”, “snow” and “ice”; and class 5 includes “water bodies”.

In total, 1,667 samples were then randomly selected within each unique stratum (encoded by a sequence of digits) resulting from the combinations of strata 1, strata 2 and strata 3 giving a total of 50,000 samples (see Table S1 and Figure S1 in the SI). To mitigate errors due to mis-registration and facilitate the implementation of different validation approaches including exact pixel matching or percentage agreement, an 80 × 80 m block composed of 64 cells of 10 × 10 m was generated for each sample point (see Figure S1).

### Crowdsourced data collection

**Fig. 1:**
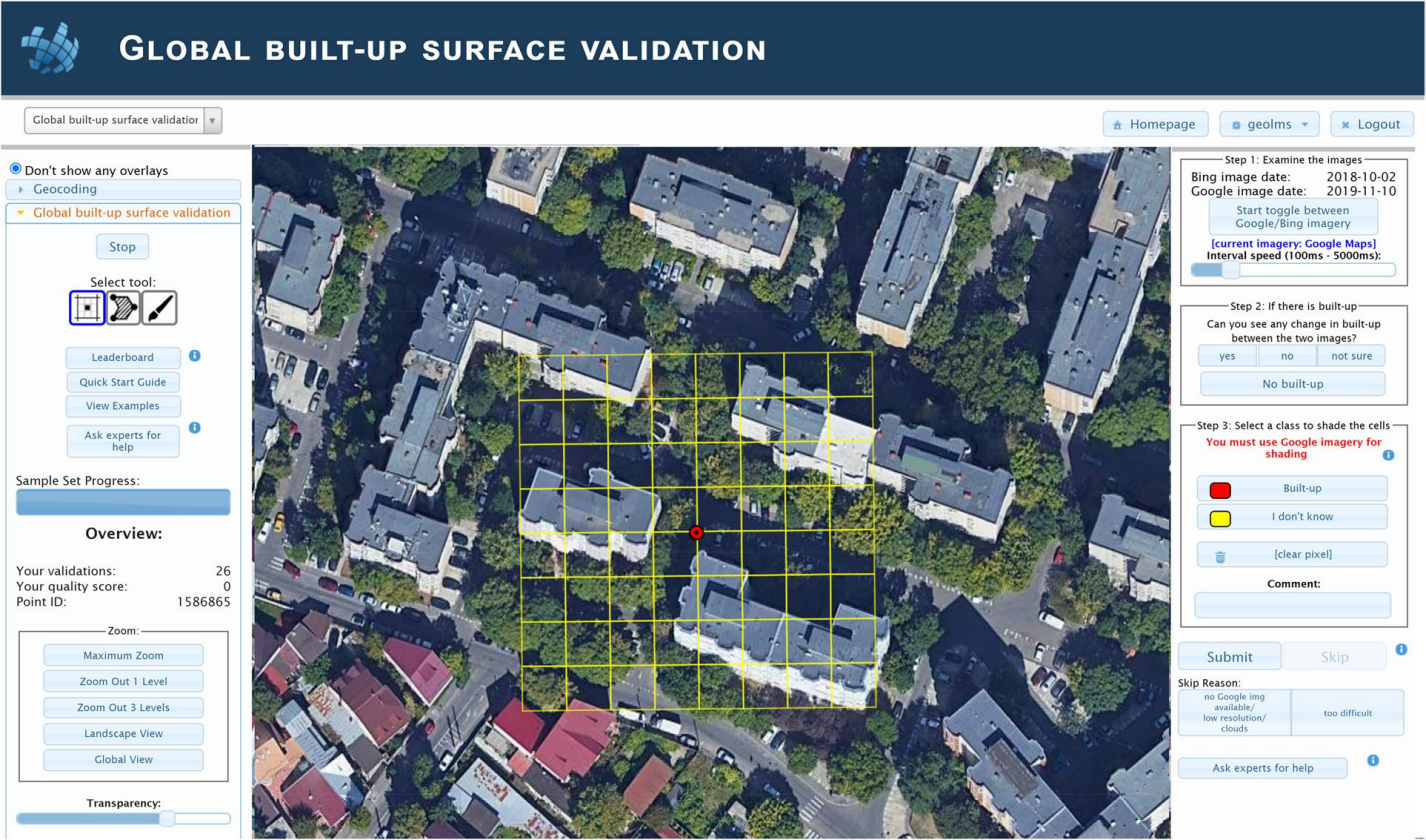
Screenshot from the Geo-Wiki Global Built-up Surface Validation branch showing an example of the data collection screen for built-up surfaces.

The built-up surface validation data were collected via a Geo-Wiki (https://www.geo-wiki.org/) campaign. Geo-Wiki is an online application for crowdsourcing the visual interpretation of very high-resolution satellite and aerial imagery, e.g., from Google Maps or Microsoft Bing Maps^24^. This application has been used in a number of data collection campaigns over the last decade, gathering data on land cover, human impact, wilderness, cropland and agricultural field size^31–33^. For this campaign we implemented a new branch of Geo‐Wiki called ‘Global Built-up Surface Validation’ as shown in Fig. 1.

**Fig. 2:**
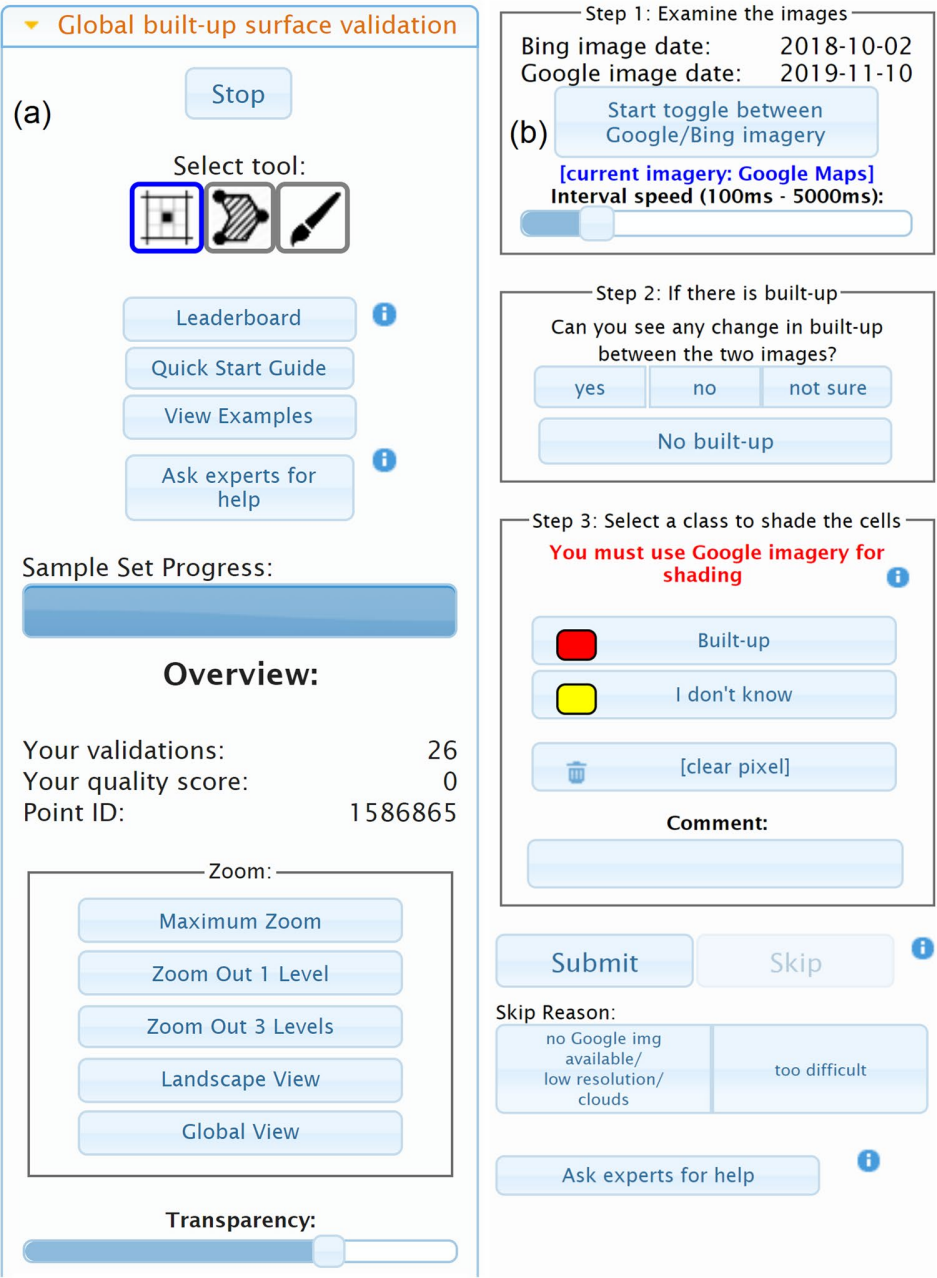
Screenshots of the (**a**) left hand and (**b**) right hand panels of the Geo-Wiki Global Built-up Surface Validation branch.

Once participants log into the application and start the validation process, they are shown a random location with a satellite or aerial image from Google Maps, overlaid with a yellow grid containing 64 cells at a resolution of 10 m each (central panel shown in Fig. 1). The user was then asked to carry out three tasks (steps 1 to 3) shown in the panel on the right of Fig. 1 and expanded to show more details in Fig. 2b.

In step 1, the user toggled between the images from Google Maps and Microsoft Bing Maps (which have different dates as shown in the step 1 box in Fig. 2b). By moving between the images, the idea was for users to a) look for presence of built-up and b) look for change in built-up between the two images, e.g., the appearance of a new building (or the disappearance of a building). The users could adjust the speed at which the images toggled back and forth, and once the animation was stopped, it would always show the Google Maps satellite image. In step 2, the user was asked to indicate if there was change in built-up, no change or to select ‘not sure’ if the change was difficult to identify. If no built-up was present, the user pressed the No-built-up button in the step 2 box (Fig. 2b), which completed the validation, and the next image was then displayed. However, if built-up was present, the user was asked to complete step 3. Users first needed to click on the Built-up button and then select the cells containing any amount of built-up in the cell, no matter how small. Different tools for selection were provided (icons located at the top of the panel shown in Fig. 2a under ‘Select tool:’), i.e., clicking on individual cells, painting areas by holding down the left mouse button or drawing a polygon around an area. Where it was difficult to determine if a building edge was in a cell, users were encouraged to select the class ‘I don’t know’ to indicate uncertainty. Comments could also be added if the user felt there was something notable in the image although we asked participants to use the comment box to tell us when imagery from Microsoft Bing was missing or if the location was completely water, e.g., in the middle of a lake. Finally, users could select a skip reason, which included situations where either imagery from Google Maps was missing, the image was obscured by clouds, it was too low in resolution (e.g., Landsat imagery) or if the image was too difficult to interpret. Once a skip reason was chosen, this would enable the Skip button, and users could then finish the validation by pressing Skip, after which the next randomly selected location would be shown.

Before starting the campaign, the participants were offered two different types of training materials: a short video explaining the purpose of the campaign and some of the functionality available from the campaign website; and a QuickStart guide which appeared when a validation session was started, which contained a series of instructions about the three tasks to be undertaken for each validation point. This QuickStart guide was also accessible at any point by clicking the appropriate button located in the left-hand panel of the Geo-Wiki interface (Fig. 2a). The ‘Ask experts for help’ button (shown on both panels in Fig. 2) generated an automated email with the point ID that went to a set of experts, who provided feedback within 24 hours to participants regarding a specific location or query.

The campaign ran during the last week of September 2020 and lasted 7 days. This was the time needed to complete the visual interpretation of 50 K points with a minimum of five times each by different participants. Communication with participants took the form of messages posted to a Geo-Wiki facebook page, via Messenger within facebook and via email. The campaign involved a broad group of participants, mainly people from universities and research institutes in the fields of remote sensing, geography and other spatial/natural sciences. See the SI for more details of the participants who filled in a survey at the end of the campaign as well as their education/expertise (Figures S2 and S3; Tables S2 and S3). The data collected during the campaign were exported from the Geo-Wiki application and made available in IIASA’s PURE repository (http://pure.iiasa.ac.at/id/eprint/17534/)^34^; the data set is described in more detail in the *Data Records* section.

### Geo-registration errors

One of the reasons why an 8 × 8 grid of 10 m cells was chosen was to account for potential geo-registration errors in the satellite and aerial imagery in Google Maps and Microsoft Bing Maps. Geo-Wiki was originally designed with an additional question regarding whether a shift was noticeable between pairs of images at the same validation location between Google Maps and Microsoft Bing Maps. However, in collecting the expert control points, we observed that small shifts were generally present but that they were usually less than 5 m in size and almost always less than one cell or 10 m in size. Moreover, shifts were sometimes difficult to detect because of differences in sensor angle between the pairs of images. Hence, we decided that this was not a good use of crowd time to record this information. Instead, we consulted the literature on studies that have investigated geo-registration errors in Google Earth. Based on a study undertaken by Paredes-Hernández *et al*.^35^, the average horizontal positional accuracy in rural areas was found to be 4.1 m, which reduced to 3.4 m when considering only imagery after 2008, which is generally the case in this campaign. Other studies cited in^35^ had higher errors but the authors criticized most of the previous studies due to potential inaccuracies in the references points or due to lack of information regarding how the errors were calculated. In the *Usage Notes*, we provide different validation modes, most of which will minimize potential errors due to geo-registration.

### Incentives and quality control during the campaign

The crowdsourcing incentives and the quality control mechanisms used in Geo-Wiki campaigns are highly interconnected. Each Geo-Wiki campaign has drawn upon previous campaign experiences, but most have the same two components that appear to work effectively. The first component is the use of prizes and co-authorship as incentives to participate. In this campaign, the top 30 participants received a prize in the form of co-authorship on this paper to recognize their contributions in gathering the data, an Amazon voucher or a combination of these two. All campaign participant co-authors were also required to provide feedback on this paper. Given that many have academic backgrounds (see Figure S2 in the SI for more details), this feedback was rich and valuable. This scientific input from participants is more akin to citizen science than crowdsourcing. Moreover, the results from the surveys administered at the end of the campaign indicated that prizes (Amazon vouchers) and co-authorship were some of the highly ranked motivations for taking part (Table S3).

**Fig. 3:**
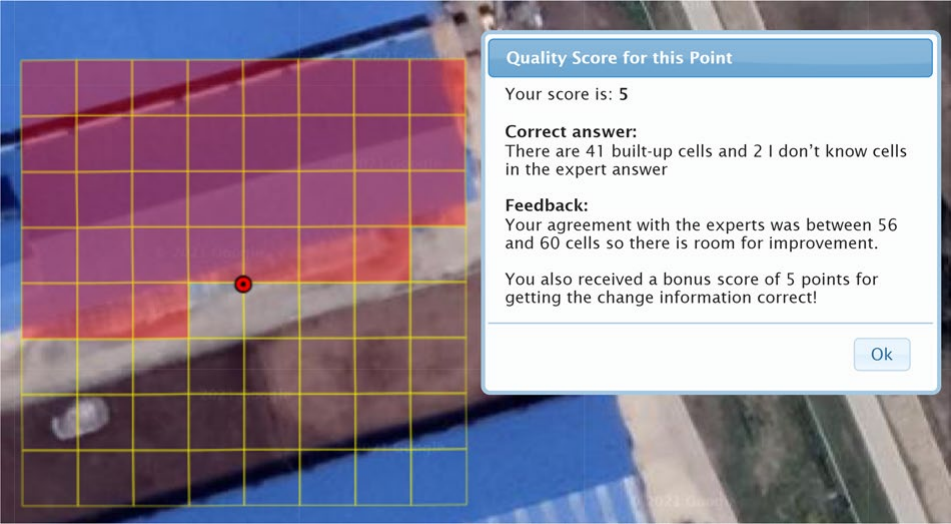
An example of the feedback provided on a control point.

The second component was the use of control points to assess quality. Control points are grids selected from the sample set that have been previously interpreted by two experts, i.e., the first and second author of this paper, both of whom have considerable experience in visual interpretation. At the start of the campaign, the participants were asked to classify 10 control points, which were chosen to illustrate different examples of non-built-up and built-up landscapes. For each sample grid classified, the participant received text‐based feedback with a score as well as the correct answer; an example is shown in Fig. 3.

After completing the 10 control points, the scores of the participants were reset to zero. From that point onward, control points were randomly shown to participants at a rate of two control points in every 20 points in the sample. Control points were the main way in which participants could increase their scores during the campaign, with maximum penalties of −35 and a maximum achievable score of +25 per control point. Details of the scoring system are provided in the SI.

The control points were chosen from the 50 K sample, originally with a proportion of 70% built-up and 30% non-built-up. However, by day 3 of the campaign, it became clear that some participants were only increasing their scores by providing answers to non-built up sample points and refreshing their browsers on built-up points to avoid the penalties. To discourage this behavior, control points with non-built-up areas were removed from the competition, scores for non-built-up control points were decreased and scoring was then only possible through visual interpretation of built-up areas. This technological design flaw in the Geo-Wiki interface was identified during this campaign, with lessons learned being carried forward to future campaigns.

## Data Records

We provide one data record (10.22022/asa/09-2021.128)^34^ with the following seven items, each of which is provided as a comma-separated file (.csv):

1. Data collected in the campaign summarized by the centroid of each grid location (Geo-WikiBuilt-upCentroidsAll.csv), in total 277,524 records.
2. Control points utilized in the campaign summarized by the centroid of each grid location (Geo-WikiBuilt-upCentroidsControls.csv), in total 1,858 records.
3. Data collected in the campaign by individual cells at each grid location (Geo-WikiBuilt-upCellsAll.csv), in total 16,765,869 records.
4. Control points utilized in the campaign by individual cells at each grid location (Geo-WikiBuilt-upCellsControls.csv), in total 118,846 records.
5. The mapping between the grid location (column: pointid) and the sampling stratum (column: stratum) (Strata.csv), in total 50,010 records.
6. Quality controlled file on change in built-up by the centroid of each grid location (Geo-WikiBuilt-upCentroidsChangeQualityControlled.csv), in total 50,010 records.
7. Quality controlled file on individual cells at each grid location (Geo-WikiBuilt-upCellsQualityControlled.csv), in total 3,200,640 records.

**Fig. 4:**
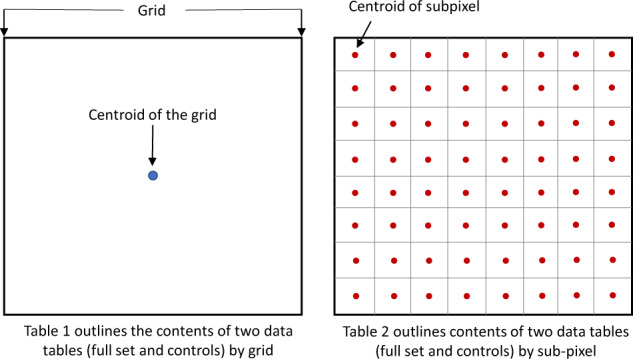
Schematic showing how the items in the data record are organized by grid and sub-pixel.

Figure 4 provides an overview of how the data are organized by grid (Table 1) and sub-pixel (Table 2) for the first four items in the data record. Tables 1 and 2 contain a list of the attributes in the first four items and their descriptions.

**Table 1:** The attributes and descriptions associated with the first two items in the data record.

Attribute	Description
SubmissionID	Unique ID of the submission
PointID	Unique ID of the grid from 1 to 50000
UserID	Unique ID of the user
Timestamp	Date and time at which the validation was made
X_Centroid	The longitude of the centroid of the 8 × 8 grid
Y_Centroid	The latitude of the centroid of the 8 × 8 grid
BingImageDate	Date of the Microsoft Bing image
GoogleImageDate	Date of the Google Maps image
*ControlPoint	Yes
	No
Legend	Change
LegendItemID	4080 = Yes
	4081 = No
	4082 = Not sure
LegendItemName	Yes
	No
	Not sure
	Null (if skipped)
*QualityofChange	100 = agreement with majority (if non-control point) or the control/includes not sure as majority (and yes/no answers)
	50 = majority was split between change and no-change (if non-control point)
	0 = no agreement with the majority (if non-control point) or the control
	Null = not applicable if a non-built-up area or skipped
BuiltupCells	The number of cells that are built-up (from 0 to 64) or null if skipped
NonBuiltupCells	The number of cells that are not built-up (from 0 to 64) or null if skipped
DoNotKnowCells	The number of cells that are marked as ‘I don’t know’ (from 0 to 64) or null if skipped
SkipReason	0 = not skipped
	1 = skipped because no Google imagery, low resolution or clouds
	2 = skipped because too difficult
*QualityofAnswer	100 = agreement with majority (if non-control point) or the control over built-up/non-built-up
	50 = majority was split between built-up/non-built up
	0 = no agreement with majority (if non-control point) or the control over built-up/non-built-up
	Null = skipped
Comment	Text-based comment about the location if present or null (as this was optional)

Attributes marked with an asterisk apply only to the first item (Geo-WikiBuilt-upCentroidsAll.csv).

**Table 2:** The attributes and descriptions associated with items 3 and 4 in the data record.

Attribute	Description
SubmissionID	Unique ID for the submission associated with a grid
SubmissionItemID	Unique ID for the submission item, i.e., unique for each individual cell
UserID	Unique ID of the user
PointID	Unique ID of the grid from 1 to 50000
SubpixelID	Unique ID of the individual cell in a grid
Legend	Built-up
LegendItemID	4002 = Built-up
	4006 = Not built-up
	4089 = I don’t know
LegendItemName	Built-up
	Not built-up
	I don’t know
SubpixelX_Centroid	The longitude of the centroid of the individual cell in the grid
SubpixelY_Centroid	The latitude of the centroid of the individual cell in the grid

Note that the sum of BuiltupCells, NonBuiltupCells and DoNotKnowCells will be 64, corresponding to the 8 × 8 grid. The three highlighted rows in Table 1 apply only to the first item in the data record (Geo-WikiBuilt-upCentroidsAll.csv), where these fields were added to provide quality control to the data set. The ControlPoint field indicates whether the answer from a participant corresponds to a control point. This information is used in the QualityofChange and QualityofAnswer fields, where values of 100 are given when the answer provided by the participant agrees either with the control point (if ControlPoint is yes) or the majority answer from the participants for that location. Disagreement is denoted by 0 while 50 has been assigned if there is no clear majority. In addition to providing information about quality, the data in these rows have also been used in the development of items 6 and 7 in the data record (see description below with Tables 3 and 4, respectively).

**Table 3:** The attributes and descriptions associated with item 6 in the data record.

Attribute	Description
PointID	Unique ID of the grid from 1 to 50000
ControlPoint	Yes
	No
Legend	Change
LegendItemID	4080 = Yes
	4081 = No
	4082 = Not sure
LegendItemName	Yes
	No
	Not sure
	Null (if skipped)
SkipReasonMajority	0 = all not skipped
	1 = majority not skipped
	2 = majority skipped
	3 = no majority

**Table 4:** The attributes and descriptions associated with item 7 in the data record.

Attribute	Description
PointID	Unique ID of the grid from 1 to 50000
SubpixelID	Unique ID of the individual cell in a grid
ControlPoint	Yes
	No
Legend	Built-up
LegendItemID	4002 = Built-up
	4006 = Not built-up
	4089 = I don’t know
LegendItemName	Built-up
	Not built-up
	I don’t know
SubpixelX_Centroid	The longitude of the centroid of the individual cell in the grid
SubpixelY_Centroid	The latitude of the centroid of the individual cell in the grid

Table 3 contains the attributes associated with item 6 in the data record, which contains quality-controlled information (Geo-WikiBuiltupCentroidsChangeQualityControlled.csv) for each location on change in built-up (if built-up is present). If the QualityofChange or the QualityofAnswer is 0 (from Table 1), then the answer is not considered further. If the point is a control point, the change information is taken from the expert. Otherwise, the change information is derived from the agreement in the answers by the participants. If there is not complete agreement, then the majority is determined and recorded. The exception is when the majority answer is No, which is then recorded as Not sure. The reason for this is that uncertainty in change is then reflected in this answer as change is much harder to detect than no change. Similarly, if the majority is split, the answer recorded is Not sure, again to reflect uncertainty.

Table 4 contains the attributes associated with item 7 in the data record, which contains quality-controlled information (Geo-WikiBuilt-upCellsQualityControlled.csv) for each location by sub-pixel. If the QualityofAnswer is 0 (from Table 1), then the answer from that participant is not considered further. If the point is a control point, the information is taken from the expert. Otherwise, the sub-pixel information is derived from the agreement in the answers provided by the participants. Similar to item 6 (change information), if there is not complete agreement for a given sub-pixel, then the majority is determined and recorded. The exception is when the majority answer is No, which is then recorded as I don’t know. The same reasoning applies as for the change information, i.e., this reflects uncertainty in the sub-pixel, e.g., sub-pixels that had a very small portion of a building in the sub-pixel, the effects of shadows, or poor-quality imagery. Similarly, if the majority is split, the answer recorded is I don’t know to reflect this uncertainty.

The data record (http://pure.iiasa.ac.at/id/eprint/17534/)^34^ also contains a shapefile with the locations of the grids and the sub-pixels. Users can spatially join the items in the data record to the shapefile to plot the information or make further analyses within a GIS (Geographic Information System) package.

**Fig. 5:**
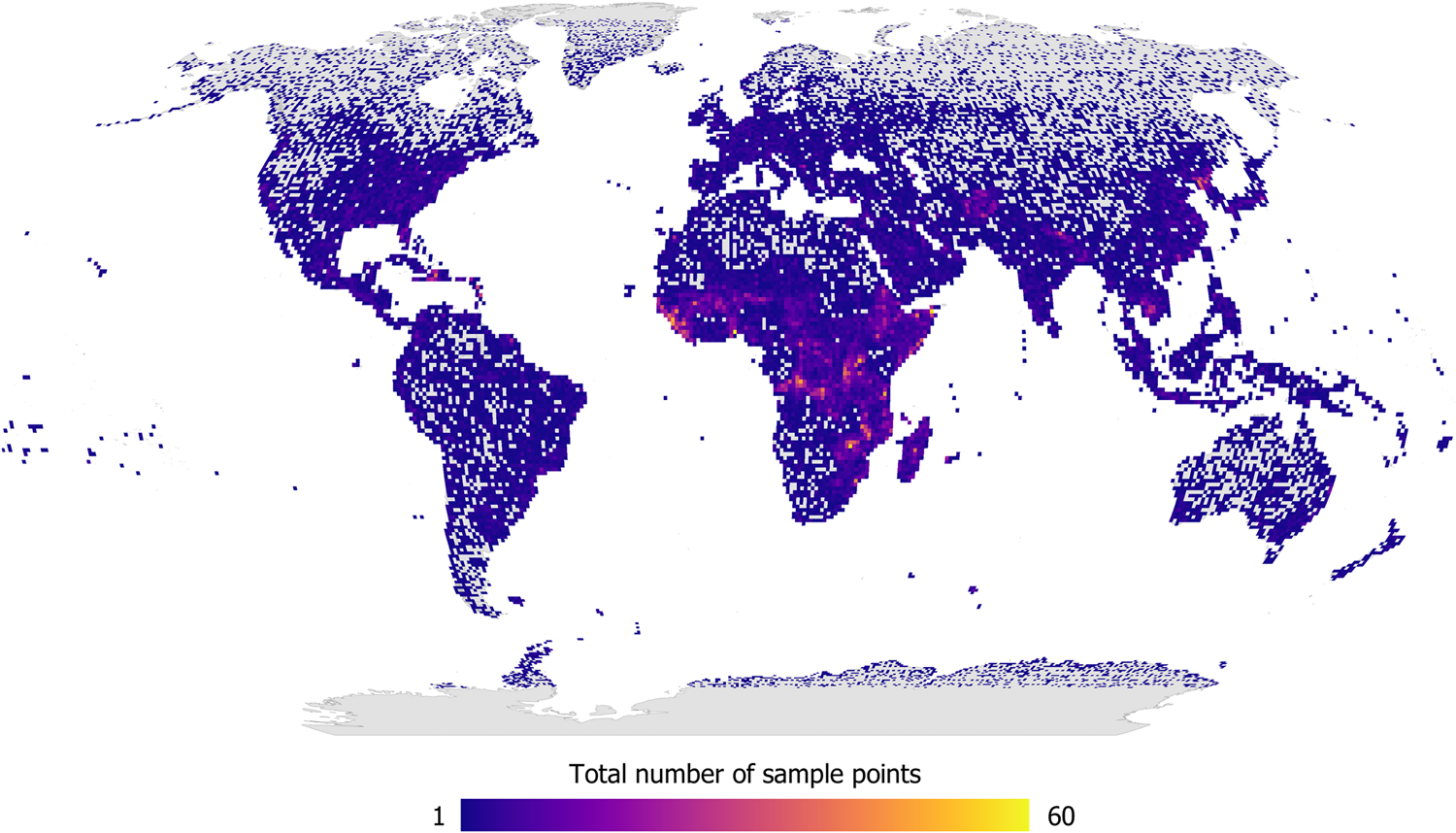
Global distribution of the sample points displayed as the total per 100 km^2^ pixels.

**Fig. 6:**
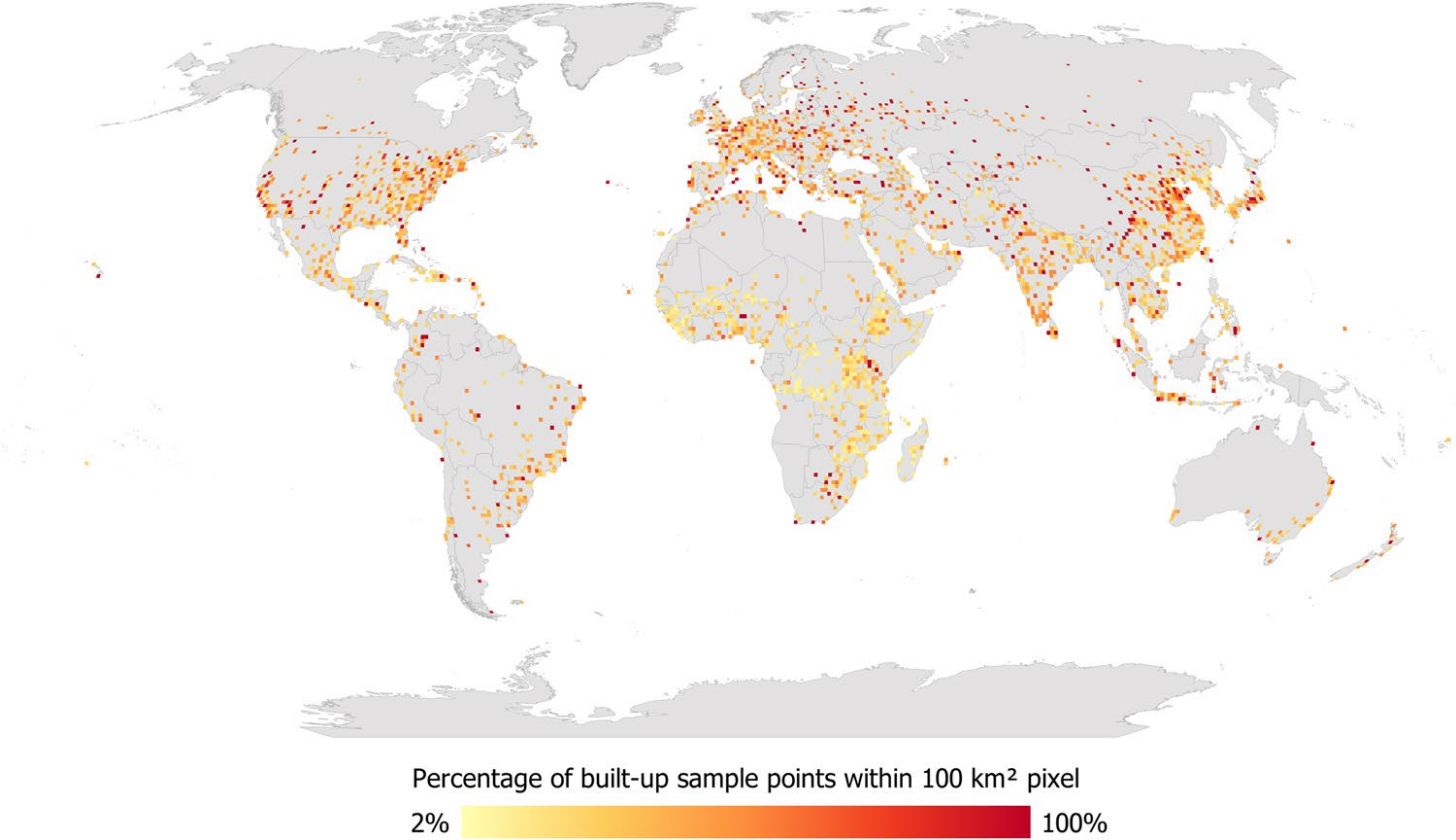
Global distribution of the built-up sample points displayed as the total by 100 km^2^ pixels.

**Fig. 7:**
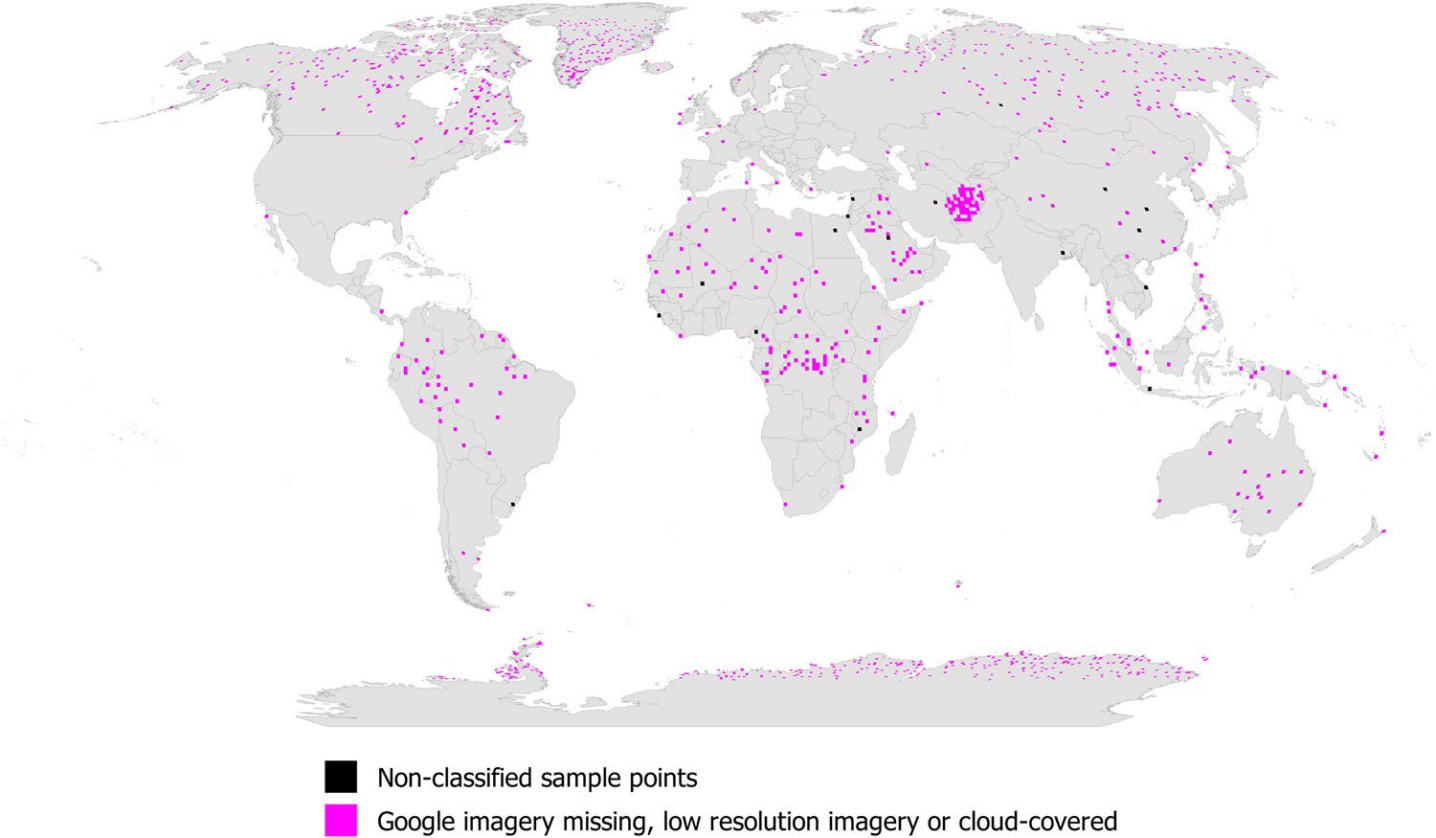
Global distribution of the sample points where imagery is missing, low resolution or cloud-covered, displayed as the total by 100 km^2^ pixels.

**Fig. 8:**
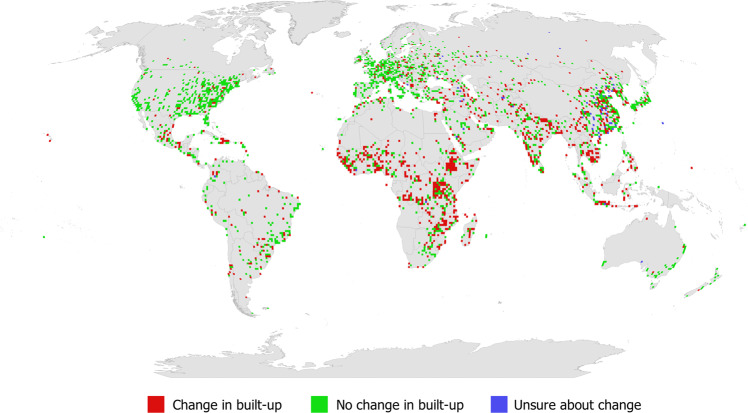
Global distribution of the built-up sample points with change information, displayed as the total by 100 km^2^ pixels.

Figure 5 shows the global distribution of sample points for the full data set collected by the participants, displayed as the number of sample points per 100 km^2^. Figure 6 shows the distribution by built-up areas (defined here as having a minimum of 1 cell of built-up in any 80 × 80 m grid) while non-built-up areas are shown in Figure S5 in the SI. Figure 7 is a map showing where Google imagery was missing, where the satellite image had too coarse a resolution for visual interpretation or where the satellite image was cloud-covered. Finally, in Fig. 8 we show built-up areas with change over time (as detected between the Google Maps and Microsoft Bing images).

## Technical Validation

Expert control points were used in the campaign to assess the quality of the participants’ classifications, calculating a score for each contribution. In total, 1,858 control points were used in the campaign, which was around 3.8% of the sample set. All the control points had a minimum of 1 cell of built-up in the grid. The control points were compared to the full set of contributions in two ways. First, the number of built-up cells per grid was reclassified into four categories in both the control and the full data set as follows:

- ≤25% built-up (1 to 16 cells)
- 25–50% built up (17 to 32 cells)
- 50–75% built-up (33 to 48 cells)
- ≥75% (49 to 64 cells)

In the first approach, the controls were compared with the full data set of participant contributions (which includes multiple contributions at the same location); these results are summarized in Table 5. The overall agreement was calculated as 76.4% while the Cohen’s kappa coefficient, which is used to measure inter-rater reliability and takes chance agreement into account^36^, was 68.8%. A Cohen’s kappa coefficient of between 0.6 and 0.8 indicates significant agreement^36^. Class agreement by row and column in Table 5 was also calculated, which summarizes the main sources of confusion between the experts and the participants. It is clear that there are situations in which the participants identified non-built-up areas while the experts found varying amounts of built-up. Moreover, class agreements are slightly lower for the classes between 25% to 75% built-up compared to those on the two extremes (less than 25% or greater than 75% urban).

**Table 5:** The comparison of expert data and all participant data summarized by categories of built-up.

		Expert control points					Class agreement (%)
		Non-built-up	<25%	25–50%	50–75%	>75%	
Participant points	Non-built-up	0	670	585	592	372	0.0
	<25%	0	6694	761	36	15	89.2
	25–50%	0	694	7540	1314	40	78.6
	50–75%	0	17	933	6240	730	78.8
	>75%	0	35	24	753	4035	83.3
Class agreement (%)		N/A	82.5	76.6	69.8	77.7	OA = 76.4%

Notes: OA is overall agreement; N/A is not applicable since control points had only built-up.

Some of these points were checked and there appear to be at least two situations in which the control point had built-up and the answer from the participant was 100% non-built-up:

1. When an expert control had very little built-up, e.g., 1 to 2 cells. In this case, it is highly likely that when working fast, the built-up areas were not seen or there is some uncertainty as to whether the cells contained built-up, so they were left as non-built-up.
2. When an expert control point had a large of amount of built-up in the grid, yet the participant chose non-built-up, which seems unlikely. Here there are two possibilities. The first is that participants did not know that certain structures were built-up, e.g., greenhouses. The second and more likely reason is related to the Geo-Wiki interface because the button for No built-up is very close to the change buttons. It was observed by the lead author and some participants that the buttons were quite sticky. Hence it was quite easy to click on No built-up when trying to click on No change. Participants complained about this during the campaign, and this may have contributed to some participants submitting a non-built-up grid when there was clearly built-up in evidence from the imagery.

To address some of the noise from those situations highlighted, the median of the built-up cells was calculated per sample point and the comparison was then undertaken; the results are provided in Table 6. The OA was higher at 84.5% while Cohen’s kappa coefficient was 78.9%.

**Table 6:** The comparison of expert data with the majority answer from participants, summarized by categories of built-up.

		Expert control points					Class agreement (%)
		Non-built-up	<25%	25–50%	50–75%	>75%	
Participant points	Non-built-up	0	3	0	1	0	0
	<25%	0	376	35	1	0	91.3
	25–50%	0	32	498	79	2	81.5
	50–75%	0	1	45	434	51	81.7
	>75%	0	0	1	37	261	84.5
Class agreement (%)		N/A	91.3	86.0	78.6	83.1	OA = 84.5%

Notes: OA is overall agreement; N/A is not applicable since control points had only built-up.

From Table 6, it is clearer to see how many sample points were completely misclassified as non-built-up compared to the expert control points, i.e., 4 sample points, which implies there is some uncertainty in these images. Table 6 also indicates other areas of large confusion, all of which should be double checked by an independent set of experts prior to use in any validation exercise.

For a more disaggregated analysis that compares expert control points with classifications from the participants, see Tables S5 to S8 in the SI, which show the same analyses as Tables 5 and 6 but for finer categories of built-up, i.e., 10 classes or a 10% interval and 20 classes or a 5% interval. The results from all three sets of analyses are summarised in Table 7. Unsurprisingly, the agreement decreases as the number of classes in the comparison increases although the use of the median always results in a higher overall accuracy. The percentage of classifications that are one class higher than the experts (overestimation) and one class lower (underestimation) are also provided in Table 7. There are tendencies towards both over and underestimation although underestimation is more prevalent. However, to put this into perspective, 20 classes or a 5% interval is equivalent to around 3 sub-pixels. Using the median as the majority approach and allowing for an over and underestimation of 3 sub-pixels results in a combined agreement of 85.8%.

**Table 7:** The comparison of expert data with answers from participants for 4, 10 and 20 classes of percentage built-up, by full agreement, percentage of classifications 1 class higher and 1 class lower than the experts, and the percentage remaining.

	Agreement (%)		One class higher than experts (%)		One class lower than experts (%)		Remaining (%)	
	All data	Majority	All data	Majority	All data	Majority	All data	Majority
4 classes (25%)	76.4	84.5	7.4	6.1	10.8	9.0	5.3	0.3
10 classes (10%)	56.3	68.1	13.9	10.9	17.4	17.3	12.4	3.7
20 classes (5%)	39.4	48.9	15.8	14.1	17.9	22.8	26.9	14.2

For non-expert control points, each location was classified five times by different participants. Due to a system error in which some observations were recorded with no values, 679 locations were classified only 4 times. To examine the consistency between participants, the agreement for built-up and non-built-up was calculated, i.e., full agreement, majority agreement or where the results were split between built-up and non-built. Tables S9 and S10 in the SI provide a summary of the agreement for all potential categories, e.g., full agreement, partial agreement, etc., and whether participants skipped a location. This information has then been aggregated and is reported in Table 8. If no skipped locations are considered, then there is full agreement between participants (considering classifications of both 4 and 5 times per location) 93.75% of the time while majority agreement is reached in 6.23% of the time. If locations with skipped answers are included (which occurs at an additional 7,127 locations, the numbers remain similar. Note that at five locations, only three classifications were made but there was full agreement. Within the full agreement category, the majority of locations were non-built-up, which indicates that non-built-up areas are generally consistently classified. For majority agreement, more of the locations were built-up, showing that there is some uncertainty over these locations, a sample of which could be checked before usage in validation.

**Table 8:** The total number of locations where there was full agreement, majority agreement and an equal split of built-up (BU) and non-built-up (NBU) without and including locations that were skipped.

Skipping	Full agreement			Majority agreement			Equal BU & NBU	Total
	BU	NBU	BU + NBU	BU	NBU	BU + NBU		
No locations skipped	565 [1.56%)	35564 [98.44%]	36129 (93.75%)	1472 [61.28%]	930 [38.72%]	2402 (6.23%)	6 (0.02%)	38537
Including locations skipped	842 [1.97%)	41986 [98.03%]	42828 (93.79%)	1735 [63.83%]	983 [36.17%]	2718 (5.95%)	118 (0.26%)	45664
Locations completely skipped	N/A	N/A	N/A	N/A	N/A	N/A	N/A	1177

N/A = not applicable.

The numbers in round brackets indicate the percentage of full and majority agreement and equal BU/NBU from the total, while numbers in square brackets indicate the percentage of BU and NBU within each category of agreement.

## Usage Notes

The data record (files Geo-WikiBuilt-upCentroidsAll.csv and Geo-WikiBuilt-upCellsAll.csv as raw data or Geo-WikiBuilt-upCellsQualityControlled.csv as consolidated quality control data) was designed to be an independent validation data set for built-up layers, which can be used in different validation modes as follows:

1. Sub-pixel matching: This would involve direct comparison of each 10 m sub-pixel with the product to be validated, either for all 64 sub-pixels or for a subset of 4, 16 or 36 sub-pixels (Fig. 9). However, this approach would be subject to geo-registration errors and is, therefore, not recommended. In addition, there are sub-pixels marked with the class ‘I don’t know’, so this would need to be handled, either by removing these or assuming that they are built-up or non-built up.

**Fig. 9:**
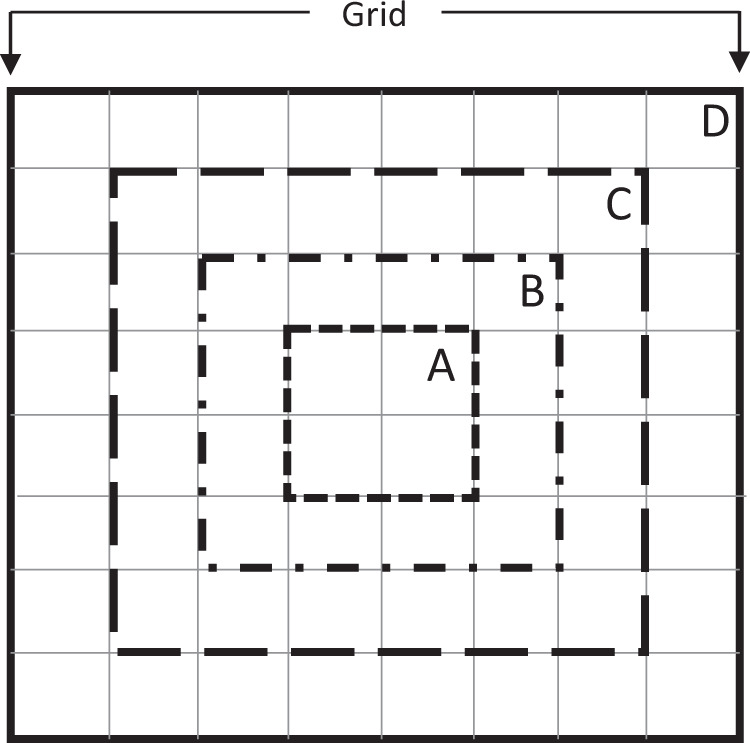
Validation modes can be applied to individual sub-pixels or blocks of sub-pixels as follows: A: 2 × 2 or 4 sub-pixels, B: 4 × 4 or 16 sub-pixels, C: 6 × 6 or 36 sub-pixels or the full grid D: 8 × 8 sub-pixels or 64 sub-pixels.
2. Block matching majority: built-up is present based on calculating the majority over 4, 16, 36 or 64 sub-pixels (Fig. 9) and setting a threshold for built-up, e.g., 50%. The class ‘I don’t know’ would need to be handled appropriately, either by removing these or assuming that they are built-up or non-built up.
3. Block matching minimum: built-up is present if a minimum of 1 sub-pixel is built-up over 4, 16, 36 or 64 sub-pixels (Fig. 9).
4. Percentage matching: the percentage of built-up can be calculated from the data record (file Geo-WikiBuilt-upCentroidsAll.csv) over 4, 16, 36, 64 sub-pixels (Fig. 9). The class ‘I don’t know’ would need to be handled appropriately.

Other validation modes would also be possible depending on the user requirements of the validation process. Moreover, it would be possible to update the data record over time using satellite imagery from Google Maps or other sources to provide an up-to-date validation data set that can be used for validating future products.

In addition to validation, the data record (files Geo-WikiBuilt-upCentroidsAll.csv and Geo-WikiBuilt-upCellsAll.csv as raw data or Geo-WikiBuilt-upCellsQualityControlled.csv as consolidated quality control data) can also be used in combination with other data sets to train algorithms in classifying the built-up class as part of broader land cover products, and for characterizing change in built-up areas. Research into the quality of crowdsourced data could be undertaken with the data record using the controls (files Geo-WikiBuilt-upCentroidsControls.csv and Geo-WikiBuilt-upCellsControls.csv) in combination with the crowdsourced data (files Geo-WikiBuilt-upCentroidsAll.csv and Geo-WikiBuilt-upCellsAll.csv).

Regarding usage of the data set for regional and national validation purposes, this would be possible by augmenting the sample^37^. For the geographical area of interest, additional sample units could be added randomly to the strata where, e.g., none are present or to increase this number. Similarly, increasing the number of samples in the built-up class could be undertaken by determining those strata in which built-up occurs more frequently and randomly allocating more sample units. As additional information, the number of points per country and per UN geographical sub-region, along with the number of points per square kilometre, are provided in Tables S11 (country) and S12 (UN geographical sub-regions).

Finally, we would like to acknowledge that the reference data set presented here is entirely produced using visual interpretation of satellite imagery using crowdsourcing, which is subjective in nature. This is not the same as using an *in-situ* ground truth data set for true validation or fully trained remote sensing experts to carry out the visual interpretation. Hence, when using the data set to calculate accuracy, this is really a measure of agreement between the visual interpretation from the crowd and the map product. However, given the new possibilities that openly available satellite imagery and crowdsourcing provide, we can now consider such an approach as a complementary way to assess the accuracy of a map product.

## Supplementary information

Supplementary Information

## Data Availability

No code has been provided because the data were analysed using ESRI’s ArcGIS software package and through queries to a postGIS database where the data were collected and stored during the Geo-Wiki campaign. The data sets are provided in comma-separated files, which can be imported into any type of analysis or GIS package for further processing.
