# Supplementary material for: A crowdsourced global data set for validating built-up surface layers
Source: Sci Data. 2022 Jan 20;9:13. doi: 10.1038/s41597-021-01105-4 (PMC8776881; doi:10.1038/s41597-021-01105-4)
Supplement: Supplementary file 1 — Supplementary Information [file 41597_2021_1105_MOESM1_ESM.docx]

**Supplementary Information for A crowdsourced global data set for validating built-up surface layers**

**Authors**

Linda See^1^, Ivelina Georgieva^1^, Martina Duerauer^1^, Thomas Kemper^2^, Christina Corbane^2^, Luca Maffenini^2^, Javier Gallego^2^, Martino Pesaresi^2^, Flavius Sirbu^3^, Rekib Ahmed^4^, Kateryna Blyshchyk^5^, Brigitte Magori^3^, Volodymyr Blyshchyk^6^, Oleksandr Melnyk^6^, Roman Zadorozhniuk^6^, Marian-Traian Mandici^7^, Yuan-Fong Su^8,9^, Ahmed Harb Rabia^10^, Ana Pérez-Hoyos^2^, Roman Vasylyshyn^6^, Chandra Kant Pawe^11^, Svitlana Bilous^6,14^, Serhii B. Kovalevskyi^6^, Sergii S. Kovalevskyi^6^, Kusumbor Bordoloi^4^, Andrii Bilous^6^, Kripal Panging^4^, Valentyn Bilous^6^, Reinhard Prestele^12^, Dhrubajyoti Sahariah^4^, Anjan Deka^4^, Nityaranjan Nath^4^, Rui Neves^13^, Viktor Myroniuk^6^, Mathias Karner^1^ and Steffen Fritz^1^

**Affiliations**

1 Ecosystem Services and Management Program, International Institute for Applied Systems Analysis (IIASA), Schlossplatz 1, Laxenburg, Austria

2 European Commission, Joint Research Center, Via Enrico Fermi, 2749, I-21027 Ispra, Italy

3 West University of Timisoara, Bulevardul Vasile Parvan no 4, Timisoara 300323

4 Department of Geography, Gauhati University, Jalukbari, Guwahati, Assam 781014, India

5 Faculty of Humanities and Pedagogy, National University of Life and Environmental Sciences of Ukraine (NULESU), Heroiv Oborony 15, Kyiv, Ukraine, 03041

6 Institute of Forestry and Landscape-Park Management, National University of Life and Environmental Sciences of Ukraine (NULESU), Heroiv Oborony 15, Kyiv, Ukraine, 03041

7 Regional Meteorological Center Banat-Crisana, Gheorghe Adam no 15, Timisoara 300310, Romania

8 Department of Harbor and River Engineering, National Taiwan Ocean University, No.2 Pei-Ning Road, Keelung, 20224, Taiwan (R.O.C.)

9 National Science and Technology Center for Disaster Reduction, 9F., No.200, Sec. 3, Beisin Rd., Xindian District, New Taipei City 23143, Taiwan (R.O.C.)

10 Damanhour University, Faculty of Agriculture, Natural Resources & Agricultural Engineering Department, El-abaadya Campus, Damanhour 22516, El-Behera, Egypt

11 Department of Geography, Pragjyotish College, Guwahati-09, Assam, India

12 Institute of Meteorology and Climate Research - Atmospheric Environmental Research (IMK-IFU), Karlsruhe Institute of Technology (KIT), Kreuzeckbahnstraße 19, 82467 Garmisch-Partenkirchen, Germany

13 Risk and Safety Department, Higher Institute of Information and Administration Sciences, Santa Joana, 3810-488 Aveiro, Portugal

14 Institute for Evolutionary Ecology, National Academy of Science of Ukraine, acad. Lebedeva, 37, Kyiv, Ukraine, 03143

Corresponding author: Linda See (see@iiasa.ac.at)

**Table of Contents**

[Background & Summary: Details of the validation of the built-up surface products (World Settlement footprint (WSF2015) and the Global Human Settlement Layer (GHSL) products 4](#_Toc81464619)

[Methods: Sampling Design 5](#_Toc81464620)

[Methods: Survey Results 6](#_Toc81464621)

[Methods: Scoring in the Global Built-up Surface Validation Campaign 8](#_Toc81464622)

[Data Records 10](#_Toc81464623)

[Technical Validation 11](#_Toc81464624)

[Comparison with Expert Control Points with More Detailed Classes 11](#_Toc81464625)

[Analysis of Consistency 15](#_Toc81464626)

[Usage Notes 17](#_Toc81464627)

**List of Figures**

[Figure S1: Spatial distribution of the sample of 50 000 points by stratum 5](#_Toc70951247)

[Figure S2: The distributions of (a) gender and (b) age (in years) of the campaign participants who filled in the survey 6](#_Toc70951248)

[Figure S3: The distributions of (a) the level of education and (b) the expertise of the campaign participants who filled in the survey 6](#_Toc70951249)

[Figure S4: (a) An expert answer, (b) a hypothetical user answer and (c) a second hypothetical user answer, where red is built-up and empty cells are non-built-up. 8](#_Toc70951250)

[Figure S5: Global distribution of the non-built-up sample points displayed as the total by 100 km^2^ pixels. 10](#_Toc70951251)

**List of Tables**

[Table S1: Three level stratification and strata design 5](#_Toc81464474)

[Table S2: The geographical distribution of campaign participants who filled in the survey 6](#_Toc81464475)

[Table S3: Ranking of motivations for participation calculated as the percentage of those participants who selected high or very high for this motivation out of all participants 7](#_Toc81464476)

[Table S4: Scoring system used in the Geo-Wiki Global Built-up Surface Validation campaign for interpretation of built-up areas 9](#_Toc81464477)

[Table S5: The comparison of expert data and all participant data summarized by categories of built-up (at 10% intervals). The diagonal (agreement) is shaded in grey while blue and orange shading denotes the number of locations where participants overestimated and underestimated values by one class, respectively. 11](#_Toc81464478)

[Table S6: The comparison of expert data with the median answer from participants, summarized by categories of built-up (at 10% intervals). The diagonal (agreement) is shaded in grey while blue and orange shading denotes the number of locations where participants overestimated and underestimated values by one class, respectively. 12](#_Toc81464479)

[Table S7: The comparison of expert data and all participant data summarized by categories of built-up (at 5% intervals). The diagonal (agreement) is shaded in grey while blue and orange shading denotes the number of locations where participants overestimated and underestimated values by one class, respectively. 13](#_Toc81464480)

[Table S8: The comparison of expert data with the majority answer from participants, summarized by categories of built-up (at 5% intervals). The diagonal (agreement) is shaded in grey while blue and orange shading denotes the number of locations where participants overestimated and underestimated values by one class, respectively. 14](#_Toc81464481)

[Table S9: The agreement of the volunteers at a location for built-up and non-built-up categories by number of times a location was skipped when a location was classified five times. 15](#_Toc81464482)

[Table S10: The agreement of the volunteers at a location for built-up and non-built-up categories by number of times a location was skipped when a location was classified four times. 15](#_Toc81464483)

[Table S11: Number of points and density per country 17](#_Toc81464484)

[Table S12: Number of points and density per geographical sub-region 22](#_Toc81464485)

# Background & Summary: Details of the validation of the built-up surface products (World Settlement footprint (WSF2015) and the Global Human Settlement Layer (GHSL) products

Based on stratified random sampling, the overall accuracy of the World Settlement Footprint (WSF2015 - considering only buildings) varies between 83.3 to 89.4%^1^. In contrast, the Global Human Settlement Layer (GHSL) Landsat-based built-up area grids (v.2015 and v.2017) and the Sentinel-1 built up area grid 2016 were validated using the GUF_DLR_v01 as a reference to document improvements in the accuracy of the products over time. The validation of the most up-to-date built-up grid derived from Sentinel-2 (Global Human Settlement Layer from JRC) considers detailed building footprints as a reference, covering 277 sites around the globe. Although this approach enables an accurate quality assessment with respect to digital cartography at a nominal scale of 1:10,000, the unbalanced spatial distribution of the sites with few reference data in Africa and Asia does not allow a comprehensive analysis of the quality of the built-up grid to be undertaken.

**References**

1. Marconcini, M. *et al.* Outlining where humans live, the World Settlement Footprint 2015. *Scientific Data* **7**, 242 (2020).

# Methods: Sampling Design

Table S1: Three level stratification and strata design


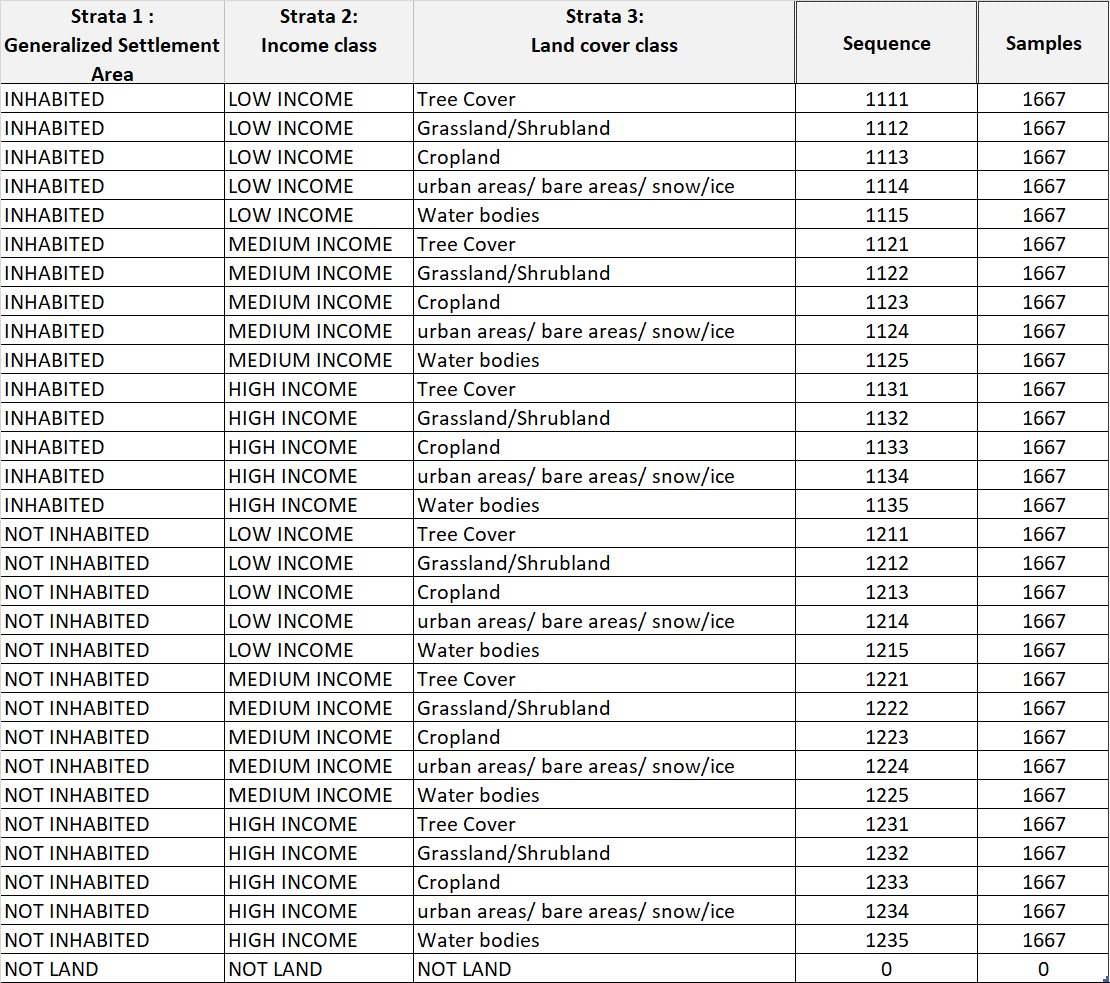


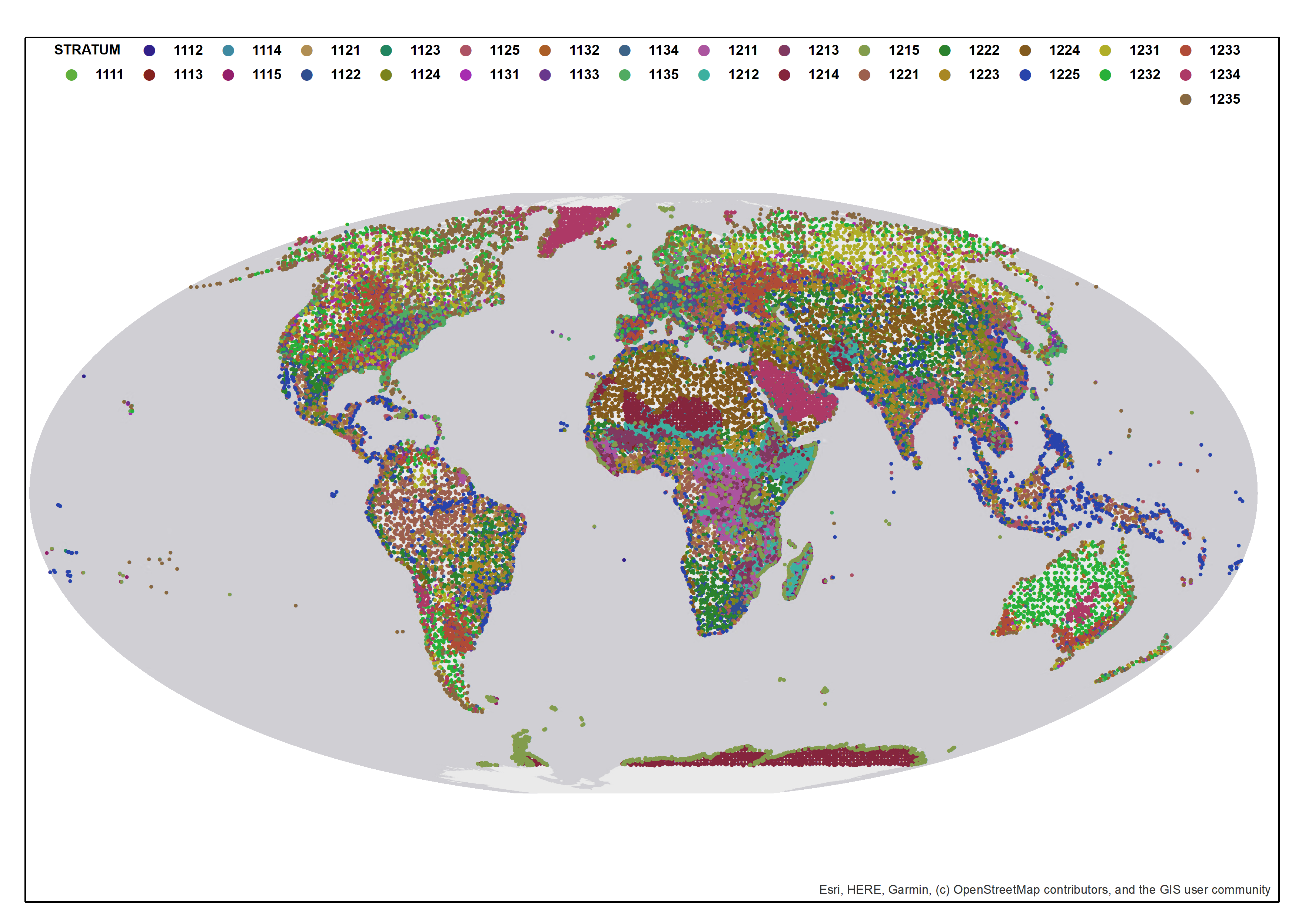


Figure S1: Spatial distribution of the sample of 50 000 points by stratum

# Methods: Survey Results

Below is summary of the results from a survey administered to participants who took part in the Geo-Wiki Global Built-up Surface Validation campaign summarize in Figures S2 and S3 and Tables S2 and S3. In total, 61 people participated in the campaign with 35 filling out the survey at the end.

| 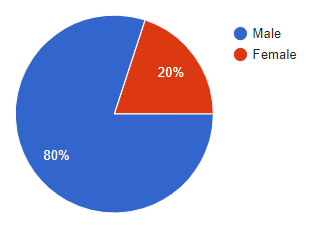  (a) | 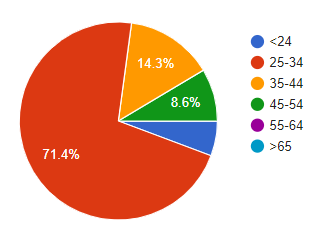  (b) |
| --- | --- |

Figure S2: The distributions of (a) gender and (b) age (in years) of the campaign participants who filled in the survey

| 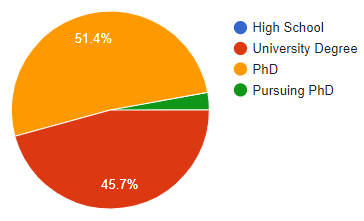  (a) | 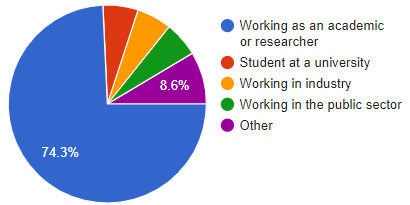  (b) |
| --- | --- |

Figure S3: The distributions of (a) the level of education and (b) the expertise of the campaign participants who filled in the survey

Table S2: The geographical distribution of campaign participants who filled in the survey

| **Country** | **Number of participants** |
| --- | --- |
| India | 12 |
| Ukraine | 12 |
| Romania | 5 |
| Italy | 2 |
| Egypt | 1 |
| Germany | 1 |
| Portugal | 1 |
| Taiwan | 1 |

Table S3: Ranking of motivations for participation calculated as the percentage of those participants who selected high or very high for this motivation out of all participants

| **Motivation** | **% selecting high or very high** |
| --- | --- |
| Happy to contribute to scientific research | 91.4 |
| This is a very interesting project! | 82.9 |
| Co-authorship | 82.9 |
| To join the Geo-Wiki community | 80.0 |
| Having fun while exploring landscapes from above | 65.7 |
| Amazon voucher | 65.7 |
| Competing against others | 48.6 |

# Methods: Scoring in the Global Built-up Surface Validation Campaign

An explanation of how the scoring worked in the campaign is provided below. Figure S4a provides an example of an expert answer while Figure S4b is a hypothetical user answer.

| \|  \|  \|  \|  \|  \|  \|  \|  \| \| --- \| --- \| --- \| --- \| --- \| --- \| --- \| --- \| \|  \|  \|  \|  \|  \|  \|  \|  \| \|  \|  \|  \|  \|  \|  \|  \|  \| \|  \|  \|  \|  \|  \|  \|  \|  \| \|  \|  \|  \|  \|  \|  \|  \|  \| \|  \|  \|  \|  \|  \|  \|  \|  \| \|  \|  \|  \|  \|  \|  \|  \|  \| \|  \|  \|  \|  \|  \|  \|  \|  \| | \|  \|  \|  \|  \|  \|  \|  \|  \| \| --- \| --- \| --- \| --- \| --- \| --- \| --- \| --- \| \|  \|  \|  \|  \|  \|  \|  \|  \| \|  \|  \|  \|  \|  \|  \|  \|  \| \|  \|  \|  \|  \|  \|  \|  \|  \| \|  \|  \|  \|  \|  \|  \|  \|  \| \|  \|  \|  \|  \|  \|  \|  \|  \| \|  \|  \|  \|  \|  \|  \|  \|  \| \|  \|  \|  \|  \|  \|  \|  \|  \| | \|  \|  \|  \|  \|  \|  \|  \|  \| \| --- \| --- \| --- \| --- \| --- \| --- \| --- \| --- \| \|  \|  \|  \|  \|  \|  \|  \|  \| \|  \|  \|  \|  \|  \|  \|  \|  \| \|  \|  \|  \|  \|  \|  \|  \|  \| \|  \|  \|  \|  \|  \|  \|  \|  \| \|  \|  \|  \|  \|  \|  \|  \|  \| \|  \|  \|  \|  \|  \|  \|  \|  \| \|  \|  \|  \|  \|  \|  \|  \|  \| |
| --- | --- | --- | --- | --- | --- | --- | --- | --- | --- | --- | --- | --- | --- | --- | --- | --- | --- | --- | --- | --- | --- | --- | --- | --- | --- | --- | --- | --- | --- | --- | --- | --- | --- | --- | --- | --- | --- | --- | --- | --- | --- | --- | --- | --- | --- | --- | --- | --- | --- | --- | --- | --- | --- | --- | --- | --- | --- | --- | --- | --- | --- | --- | --- | --- | --- | --- | --- | --- | --- | --- | --- | --- | --- | --- | --- | --- | --- | --- | --- | --- | --- | --- | --- | --- | --- | --- | --- | --- | --- | --- | --- | --- | --- | --- | --- | --- | --- | --- | --- | --- | --- | --- | --- | --- | --- | --- | --- | --- | --- | --- | --- | --- | --- | --- | --- | --- | --- | --- | --- | --- | --- | --- | --- | --- | --- | --- | --- | --- | --- | --- | --- | --- | --- | --- | --- | --- | --- | --- | --- | --- | --- | --- | --- | --- | --- | --- | --- | --- | --- | --- | --- | --- | --- | --- | --- | --- | --- | --- | --- | --- | --- | --- | --- | --- | --- | --- | --- | --- | --- | --- | --- | --- | --- | --- | --- | --- | --- | --- | --- | --- | --- | --- | --- | --- | --- | --- | --- | --- | --- | --- | --- | --- | --- | --- |
| (a) | (b) | (c) |

Figure S4: (a) An expert answer, (b) a hypothetical user answer and (c) a second hypothetical user answer, where red is built-up and empty cells are non-built-up.

We start with the cell in row 1, column 1. They are both non-built-up so the agreement between the expert and the user is 1. We move to row 1, column 2 and there is agreement once again, so the total agreement is then 2. We repeat this for all 64 cells. In this case the agreement is 63 since the user said non-built-up in 1 cell where the expert said built-up (row 4, column 5). For this answer, the user would get a perfect score of 20 because we allow for a mistake of 1 cell.

Take the second hypothetical user answer (Figure S4c), the agreement would be 61 because two further built-up cells labelled by the user do not appear in the expert answer. For this answer, the participant would receive 10 points because we allow for between 2 to 3 mistakes to achieve this score.

Table S4 provides the range of scores with an explanation of how they were assigned. However, it was also possible to receive a score of -30, but this was only applied in extreme cases where the participant did one of two things:

1. Shaded all cells as built-up in a complex landscape where some cells are non-urban.
2. Shaded no cells as built-up in a landscape where only a few cells are urban.

This latter scoring decision was based on an observed participant behaviour from a previous campaign where participants did not shade cropland when only a few cropland cells were presented and shaded all cropland when only a few cells were non-cropland because it was faster to do this and they still received high scores. Hence, high penalties were given in this campaign for these two cases.

In addition to scores for shading the grid, if the change question was answered correctly, participants received 5 additional points. If the change information was wrong, participants lost 5 points. If the expert was unsure about the change in built-up between the images from Google Maps and Microsoft Bing Maps, then no points were awarded for the change information provided by the participant, regardless of their answer.

A maximum penalty of -35 could be received in the situation described above where -30 was assigned and the change information was incorrect, incurring an addition -5. The maximum score that could be achieved was +25 in which the shading of cells was correct (+20) and the change information was also correct (+5).

Table S4: Scoring system used in the Geo-Wiki Global Built-up Surface Validation campaign for interpretation of built-up areas

| **Score** | **Feedback** | **Number of allowable mistakes** |
| --- | --- | --- |
| 20 | Congratulations! You got a perfect score for identifying the built-up/non-built up areas! | 0 to 1 cells are wrong |
| 10 | You were close to agreeing with the experts but got a few cells wrong. | 2 to 3 cells are wrong |
| 0 | Your agreement with the experts was between 56 and 60 cells so there is room for improvement. | 4 to 8 cells are wrong |
| -10 | Your agreement with the experts was between 40 and 55 cells so there is room for improvement. | 9 to 24 cells are wrong |
| -20 | Your agreement with the experts was less than 40 cells so there is room for improvement. | 25 or more cells are wrong |

Finally, if the expert shaded a grid cell as ‘I don’t know’, this is treated as agreement, regardless of the answer provided by the participant. This is because we did not wish to penalize participants for cells that contained some element of uncertainty, e.g., a tiny section of a building where it was not clear if it really fell in the cell or an object that might be a building but was difficult to see due to the quality of the image.

In some cases, the participants queried the expert control points by clicking on the ‘Ask the experts’ button located within the Geo-Wiki interface. This sent an email to the Geo-Wiki team, who then checked the control point. Where the participant was correct and there was an error in the control point, which did occur in some cases, this point was fixed, rewritten to the database and the scores of the participant were adjusted.

# Data Records


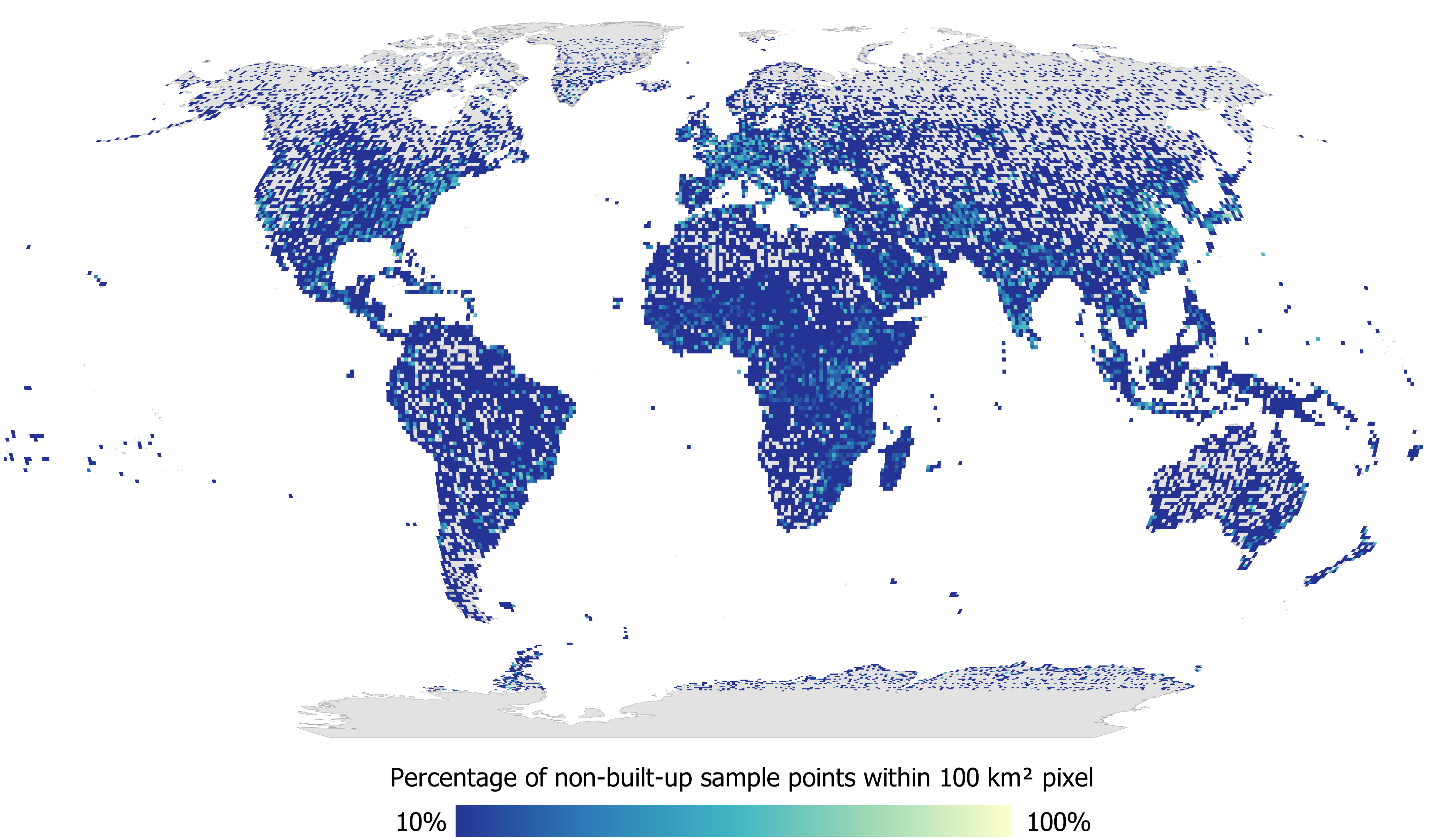


Figure S5: Global distribution of the non-built-up sample points displayed as the total by 100 km^2^ pixels.

# Technical Validation

## Comparison with Expert Control Points with More Detailed Classes

Additional analyses were run for a finer set of classes than 25% intervals. Table S5 shows the comparison between expert control points and the full set of participant contributions (which includes multiple contributions at the same location) when the number of built-up cells per grid was reclassified into ten classes or intervals of 10%. To address some of the noise arising from those situations highlighted in the main paper, the median of the built-up cells was calculated per sample point and the comparison was then undertaken where the results are provided in Table S6.

Table S5: The comparison of expert data and all participant data summarized by categories of built-up (at 10% intervals). The diagonal (agreement) is shaded in grey while blue and orange shading denotes the number of locations where participants overestimated and underestimated values by one class, respectively.

|  | | Expert control points | | | | | | | | | | | Class agreement (%) |
| --- | --- | --- | --- | --- | --- | --- | --- | --- | --- | --- | --- | --- | --- |
|  |  | Non-built-up | <10% | 10 to <20% | 20 to < 30% | 30 to < 40% | 40 to < 50% | 50 to < 60% | 60 to < 70% | 70 to < 80% | 80 to < 90% | 90 to 100% |  |
| Participant points | Non-built-up | 0 | 379 | 156 | 226 | 202 | 292 | 239 | 227 | 215 | 154 | 129 | 0.0 |
|  | <10% | 0 | 2394 | 269 | 18 | 4 | 10 | 0 | 3 | 0 | 1 | 2 | 88.6 |
|  | 10 to <20% | 0 | 338 | 1611 | 536 | 59 | 18 | 7 | 7 | 2 | 1 | 3 | 62.4 |
|  | 20 to < 30% | 0 | 38 | 490 | 2345 | 678 | 152 | 35 | 20 | 4 | 3 | 1 | 62.3 |
|  | 30 to < 40% | 0 | 3 | 27 | 530 | 2164 | 815 | 121 | 61 | 12 | 2 | 3 | 57.9 |
|  | 40 to < 50% | 0 | 1 | 3 | 81 | 600 | 2505 | 831 | 222 | 58 | 3 | 3 | 58.2 |
|  | 50 to < 60% | 0 | 0 | 1 | 15 | 85 | 630 | 1632 | 788 | 176 | 19 | 5 | 48.7 |
|  | 60 to < 70% | 0 | 1 | 1 | 4 | 15 | 155 | 629 | 1375 | 641 | 55 | 27 | 47.4 |
|  | 70 to < 80% | 0 | 3 | 0 | 5 | 3 | 52 | 123 | 516 | 1554 | 328 | 100 | 57.9 |
|  | 80 to < 90% | 0 | 4 | 0 | 5 | 0 | 8 | 16 | 94 | 436 | 877 | 306 | 50.2 |
|  | 90 to 100% | 0 | 0 | 0 | 21 | 0 | 1 | 6 | 35 | 109 | 316 | 1595 | 76.6 |
| Class agreement (%) | | N/A | 75.7 | 63.0 | 61.9 | 56.8 | 54.0 | 44.8 | 41.1 | 48.5 | 49.9 | 73.4 | OA=56.3% |

Table S6: The comparison of expert data with the median answer from participants, summarized by categories of built-up (at 10% intervals). The diagonal (agreement) is shaded in grey while blue and orange shading denotes the number of locations where participants overestimated and underestimated values by one class, respectively.

|  | | Expert control points | | | | | | | | | | | Class agreement (%) |
| --- | --- | --- | --- | --- | --- | --- | --- | --- | --- | --- | --- | --- | --- |
|  |  | Non-built-up | <10% | 10 to <20% | 20 to < 30% | 30 to < 40% | 40 to < 50% | 50 to < 60% | 60 to < 70% | 70 to < 80% | 80 to < 90% | 90 to 100% |  |
| Participant points | Non-built-up | 0 | 3 | 0 | 0 | 0 | 0 | 0 | 1 | 0 | 0 | 0 | 0.0 |
|  | <10% | 0 | 131 | 12 | 0 | 0 | 0 | 0 | 0 | 0 | 0 | 0 | 91.6 |
|  | 10 to <20% | 0 | 14 | 100 | 25 | 0 | 0 | 0 | 0 | 0 | 0 | 0 | 71.9 |
|  | 20 to < 30% | 0 | 2 | 22 | 153 | 35 | 7 | 2 | 0 | 0 | 0 | 0 | 69.2 |
|  | 30 to < 40% | 0 | 0 | 0 | 25 | 162 | 44 | 3 | 1 | 1 | 0 | 0 | 68.6 |
|  | 40 to < 50% | 0 | 0 | 0 | 2 | 17 | 190 | 61 | 13 | 1 | 0 | 0 | 66.9 |
|  | 50 to < 60% | 0 | 0 | 0 | 0 | 4 | 32 | 123 | 49 | 7 | 0 | 1 | 56.9 |
|  | 60 to < 70% | 0 | 0 | 0 | 1 | 0 | 9 | 39 | 109 | 48 | 2 | 0 | 52.4 |
|  | 70 to < 80% | 0 | 0 | 0 | 0 | 0 | 1 | 2 | 23 | 124 | 23 | 2 | 70.9 |
|  | 80 to < 90% | 0 | 0 | 0 | 0 | 0 | 0 | 0 | 4 | 20 | 74 | 21 | 62.2 |
|  | 90 to 100% | 0 | 0 | 0 | 0 | 0 | 0 | 0 | 0 | 2 | 11 | 99 | 88.4 |
| Class agreement (%) | | 0.0 | 4.1 | 3.9 | 4.0 | 4.3 | 4.1 | 3.4 | 3.3 | 3.9 | 4.2 | 4.6 | OA=68.1% |

The above analysis was repeated for an even finer set of classes. Table S7 shows the comparison between expert control points and the full set of participant contributions (which includes multiple contributions at the same location) when the number of built-up cells per grid was reclassified into twenty classes or intervals of 5% while Table S8 provides the same analysis but using the median of the built-up cells per sample point for the comparison.

Table S7: The comparison of expert data and all participant data summarized by categories of built-up (at 5% intervals). The diagonal (agreement) is shaded in grey while blue and orange shading denotes the number of locations where participants overestimated and underestimated values by one class, respectively.

|  | | Expert control points | | | | | | | | | | | | | | | | | | | | | Class agreement (%) |
| --- | --- | --- | --- | --- | --- | --- | --- | --- | --- | --- | --- | --- | --- | --- | --- | --- | --- | --- | --- | --- | --- | --- | --- |
|  |  | NBU | 1 | 2 | 3 | 4 | 5 | 6 | 7 | 8 | 9 | 10 | 11 | 12 | 13 | 14 | 15 | 16 | 17 | 18 | 19 | 20 |  |
| Participant points | NBU | 0 | 218 | 161 | 57 | 99 | 135 | 91 | 108 | 94 | 116 | 176 | 130 | 109 | 121 | 106 | 126 | 89 | 74 | 80 | 47 | 82 | 0.0 |
|  | 1 | 0 | 876 | 242 | 18 | 7 | 4 | 0 | 1 | 0 | 2 | 5 | 0 | 0 | 0 | 0 | 0 | 0 | 1 | 0 | 0 | 1 | 75.7 |
|  | 2 | 0 | 146 | 1130 | 201 | 43 | 12 | 2 | 3 | 0 | 2 | 1 | 0 | 0 | 2 | 1 | 0 | 0 | 0 | 0 | 0 | 1 | 73.2 |
|  | 3 | 0 | 30 | 253 | 572 | 218 | 92 | 11 | 12 | 3 | 1 | 8 | 1 | 2 | 1 | 5 | 0 | 0 | 1 | 0 | 0 | 0 | 47.3 |
|  | 4 | 0 | 6 | 49 | 193 | 628 | 371 | 62 | 28 | 16 | 5 | 4 | 2 | 2 | 0 | 1 | 0 | 2 | 0 | 0 | 0 | 3 | 45.8 |
|  | 5 | 0 | 4 | 19 | 51 | 362 | 1167 | 330 | 157 | 60 | 29 | 19 | 14 | 1 | 2 | 2 | 0 | 2 | 2 | 1 | 0 | 1 | 52.5 |
|  | 6 | 0 | 13 | 2 | 13 | 64 | 357 | 491 | 369 | 92 | 68 | 36 | 12 | 8 | 6 | 10 | 1 | 1 | 0 | 0 | 0 | 0 | 31.8 |
|  | 7 | 0 | 1 | 2 | 3 | 18 | 122 | 265 | 799 | 323 | 163 | 73 | 23 | 11 | 10 | 11 | 3 | 1 | 0 | 0 | 0 | 0 | 43.7 |
|  | 8 | 0 | 0 | 0 | 1 | 5 | 53 | 90 | 390 | 652 | 384 | 195 | 58 | 29 | 26 | 14 | 4 | 4 | 0 | 2 | 2 | 1 | 34.1 |
|  | 9 | 0 | 0 | 0 | 0 | 3 | 23 | 30 | 119 | 293 | 609 | 477 | 141 | 71 | 42 | 20 | 10 | 14 | 0 | 0 | 0 | 0 | 32.9 |
|  | 10 | 0 | 1 | 0 | 0 | 0 | 13 | 15 | 44 | 144 | 358 | 1061 | 419 | 200 | 112 | 48 | 25 | 9 | 3 | 0 | 1 | 2 | 43.2 |
|  | 11 | 0 | 0 | 0 | 0 | 1 | 5 | 3 | 24 | 33 | 82 | 362 | 460 | 354 | 193 | 74 | 37 | 18 | 2 | 4 | 1 | 2 | 27.8 |
|  | 12 | 0 | 0 | 0 | 0 | 0 | 5 | 2 | 12 | 16 | 28 | 158 | 281 | 537 | 372 | 149 | 89 | 32 | 11 | 2 | 1 | 1 | 31.7 |
|  | 13 | 0 | 0 | 0 | 0 | 1 | 1 | 0 | 2 | 10 | 12 | 96 | 163 | 278 | 443 | 247 | 161 | 46 | 15 | 6 | 9 | 4 | 29.7 |
|  | 14 | 0 | 0 | 1 | 0 | 0 | 2 | 1 | 0 | 3 | 14 | 33 | 69 | 119 | 261 | 424 | 326 | 108 | 21 | 13 | 10 | 4 | 30.1 |
|  | 15 | 0 | 0 | 0 | 0 | 0 | 1 | 1 | 3 | 0 | 11 | 27 | 40 | 69 | 138 | 275 | 681 | 249 | 90 | 32 | 35 | 14 | 40.9 |
|  | 16 | 0 | 3 | 0 | 0 | 0 | 3 | 0 | 0 | 0 | 3 | 11 | 3 | 11 | 24 | 79 | 264 | 360 | 152 | 54 | 37 | 14 | 35.4 |
|  | 17 | 0 | 4 | 0 | 0 | 0 | 2 | 0 | 0 | 0 | 1 | 3 | 7 | 5 | 16 | 54 | 125 | 183 | 202 | 189 | 67 | 34 | 22.6 |
|  | 18 | 0 | 0 | 0 | 0 | 0 | 3 | 0 | 0 | 0 | 3 | 1 | 3 | 1 | 1 | 23 | 45 | 83 | 150 | 336 | 136 | 69 | 39.3 |
|  | 19 | 0 | 0 | 0 | 0 | 0 | 18 | 0 | 0 | 0 | 0 | 0 | 0 | 3 | 1 | 11 | 20 | 40 | 51 | 158 | 260 | 159 | 36.1 |
|  | 20 | 0 | 0 | 0 | 0 | 0 | 2 | 1 | 0 | 0 | 1 | 0 | 0 | 3 | 12 | 11 | 31 | 18 | 32 | 75 | 230 | 946 | 69.5 |
| Class agreement (%) | | N/A | 67.3 | 60.8 | 51.6 | 43.3 | 48.8 | 35.2 | 38.6 | 37.5 | 32.2 | 38.6 | 25.2 | 29.6 | 24.8 | 27.1 | 35.0 | 28.6 | 25.0 | 35.3 | 31.1 | 70.7 | OA=39.4% |

Classes: NBU = non-built-up; 1 = < 5%; 2 = 5 to < 10%; 3 = 10 to < 15%; 4 = 15 to < 20%; 5 = 20 to < 25%; 6 = 25 to < 30%; 7 = 30 to < 35%; 8 = 35 to < 40%; 9 = 40 to < 45%; 10 = 45 to < 50%; 11 = 50 to < 55%; 12 = 55 to < 60%; 13 = 60 to < 65%; 14 = 65 to < 70%; 15 = 70 to < 75%; 16 = 75 to < 80%; 17 = 80 to < 85%; 18 = 85 to < 90%; 19 = 90 to < 95%; 20 = 95 to < 100%

Table S8: The comparison of expert data with the majority answer from participants, summarized by categories of built-up (at 5% intervals). The diagonal (agreement) is shaded in grey while blue and orange shading denotes the number of locations where participants overestimated and underestimated values by one class, respectively.

|  | | Expert control points | | | | | | | | | | | | | | | | | | | | | Class agreement (%) |
| --- | --- | --- | --- | --- | --- | --- | --- | --- | --- | --- | --- | --- | --- | --- | --- | --- | --- | --- | --- | --- | --- | --- | --- |
|  |  | NBU | 1 | 2 | 3 | 4 | 5 | 6 | 7 | 8 | 9 | 10 | 11 | 12 | 13 | 14 | 15 | 16 | 17 | 18 | 19 | 20 |  |
| Participant points | NBU | 0 | 3 | 0 | 0 | 0 | 0 | 0 | 0 | 0 | 0 | 0 | 0 | 0 | 1 | 0 | 0 | 0 | 0 | 0 | 0 | 0 | 0.0 |
|  | 1 | 0 | 52 | 8 | 0 | 0 | 0 | 0 | 0 | 0 | 0 | 0 | 0 | 0 | 0 | 0 | 0 | 0 | 0 | 0 | 0 | 0 | 86.7 |
|  | 2 | 0 | 3 | 68 | 10 | 2 | 0 | 0 | 0 | 0 | 0 | 0 | 0 | 0 | 0 | 0 | 0 | 0 | 0 | 0 | 0 | 0 | 81.9 |
|  | 3 | 0 | 2 | 11 | 33 | 12 | 2 | 0 | 0 | 0 | 0 | 0 | 0 | 0 | 0 | 0 | 0 | 0 | 0 | 0 | 0 | 0 | 55.0 |
|  | 4 | 0 | 0 | 1 | 12 | 43 | 22 | 1 | 0 | 0 | 0 | 0 | 0 | 0 | 0 | 0 | 0 | 0 | 0 | 0 | 0 | 0 | 54.4 |
|  | 5 | 0 | 0 | 1 | 2 | 16 | 76 | 19 | 7 | 3 | 2 | 3 | 1 | 0 | 0 | 0 | 0 | 0 | 0 | 0 | 0 | 0 | 58.5 |
|  | 6 | 0 | 1 | 0 | 0 | 4 | 18 | 40 | 22 | 3 | 2 | 0 | 0 | 1 | 0 | 0 | 0 | 0 | 0 | 0 | 0 | 0 | 44.0 |
|  | 7 | 0 | 0 | 0 | 0 | 0 | 4 | 14 | 61 | 20 | 4 | 1 | 0 | 0 | 0 | 0 | 0 | 0 | 0 | 0 | 0 | 0 | 58.7 |
|  | 8 | 0 | 0 | 0 | 0 | 0 | 4 | 3 | 24 | 57 | 29 | 10 | 2 | 1 | 0 | 1 | 0 | 1 | 0 | 0 | 0 | 0 | 43.2 |
|  | 9 | 0 | 0 | 0 | 0 | 0 | 1 | 1 | 3 | 11 | 52 | 38 | 7 | 3 | 3 | 0 | 0 | 1 | 0 | 0 | 0 | 0 | 43.3 |
|  | 10 | 0 | 0 | 0 | 0 | 0 | 0 | 0 | 0 | 3 | 17 | 83 | 41 | 10 | 7 | 3 | 0 | 0 | 0 | 0 | 0 | 0 | 50.6 |
|  | 11 | 0 | 0 | 0 | 0 | 0 | 0 | 0 | 1 | 2 | 2 | 19 | 35 | 34 | 8 | 6 | 1 | 0 | 0 | 0 | 0 | 0 | 32.4 |
|  | 12 | 0 | 0 | 0 | 0 | 0 | 0 | 0 | 0 | 1 | 0 | 11 | 19 | 35 | 30 | 5 | 4 | 2 | 0 | 0 | 0 | 1 | 32.4 |
|  | 13 | 0 | 0 | 0 | 0 | 0 | 0 | 0 | 0 | 0 | 1 | 4 | 8 | 25 | 41 | 24 | 6 | 3 | 1 | 0 | 0 | 0 | 36.3 |
|  | 14 | 0 | 0 | 0 | 0 | 0 | 1 | 0 | 0 | 0 | 2 | 2 | 5 | 1 | 13 | 31 | 30 | 9 | 1 | 0 | 0 | 0 | 32.6 |
|  | 15 | 0 | 0 | 0 | 0 | 0 | 0 | 0 | 0 | 0 | 0 | 0 | 0 | 2 | 4 | 11 | 56 | 23 | 8 | 2 | 1 | 0 | 52.3 |
|  | 16 | 0 | 0 | 0 | 0 | 0 | 0 | 0 | 0 | 0 | 0 | 1 | 0 | 0 | 0 | 8 | 14 | 31 | 12 | 1 | 1 | 0 | 45.6 |
|  | 17 | 0 | 0 | 0 | 0 | 0 | 0 | 0 | 0 | 0 | 0 | 0 | 0 | 0 | 0 | 4 | 9 | 6 | 15 | 18 | 3 | 2 | 26.3 |
|  | 18 | 0 | 0 | 0 | 0 | 0 | 0 | 0 | 0 | 0 | 0 | 0 | 0 | 0 | 0 | 0 | 0 | 5 | 14 | 27 | 12 | 4 | 43.5 |
|  | 19 | 0 | 0 | 0 | 0 | 0 | 0 | 0 | 0 | 0 | 0 | 0 | 0 | 0 | 0 | 0 | 1 | 0 | 2 | 5 | 22 | 16 | 47.8 |
|  | 20 | 0 | 0 | 0 | 0 | 0 | 0 | 0 | 0 | 0 | 0 | 0 | 0 | 0 | 0 | 0 | 1 | 0 | 0 | 4 | 10 | 51 | 77.3 |
| Class agreement (%) | | N/A | 85.2 | 76.4 | 57.9 | 55.8 | 59.4 | 51.3 | 51.7 | 57.0 | 46.8 | 48.3 | 29.7 | 31.3 | 38.3 | 33.3 | 45.9 | 38.3 | 28.3 | 47.4 | 44.9 | 68.9 | OA= 48.9% |

Classes: NBU = non-built-up; 1 = < 5%; 2 = 5 to < 10%; 3 = 10 to < 15%; 4 = 15 to < 20%; 5 = 20 to < 25%; 6 = 25 to < 30%; 7 = 30 to < 35%; 8 = 35 to < 40%; 9 = 40 to < 45%; 10 = 45 to < 50%; 11 = 50 to < 55%; 12 = 55 to < 60%; 13 = 60 to < 65%; 14 = 65 to < 70%; 15 = 70 to < 75%; 16 = 75 to < 80%; 17 = 80 to < 85%; 18 = 85 to < 90%; 19 = 90 to < 95%; 20 = 95 to < 100%

## Analysis of Consistency

Since each location was classified five times by the participants, the agreement between participants was calculated to evaluate the consistency of responses for each location. Since participants can skip a location due to lack of imagery, low resolution, presence of clouds or uncertainty, the agreement was summarized in Table S9 according to how many of the five locations were skipped and by agreement between participants in terms of built-up and non-built-up. Note that built-up is defined here as having a minimum of one grid cell marked as built-up.

Table S9: The agreement of the volunteers at a location for built-up and non-built-up categories by number of times a location was skipped when a location was classified five times.

|  | 5 agreed | | 4 agreed | | 3 agreed | | 2 agreed | | |
| --- | --- | --- | --- | --- | --- | --- | --- | --- | --- |
|  | Built-up | Non-built-up | Built-up | Non-built-up | Built-up | Non-built-up | Built-up | Non-built-up | Equal Built-up/  Non-built-up |
| No skipping | 547 | 35050 | 910 | 568 | 523 | 356 | N/A | N/A | N/A |
| 1 skipped | N/A | N/A | 242 | 3720 | 260 | 36 | N/A | N/A | 118 |
| 2 skipped | N/A | N/A | N/A | N/A | 33 | 2590 | 25 | 16 | N/A |
| 3 skipped | N/A | N/A | N/A | N/A | N/A | N/A | 0 | 0 | 0 |

Skipped > 3 times = 1174 locations; NA = not applicable

At some locations, participants only classified the location four times. The reason for this is that some classifications were recorded as 0 for all categories in the database, which was a system error. These observations were removed from the database. Table S10 summarizes the agreement between participants in terms of built-up and non-built for each location according to how many of the four locations were skipped.

Table S10: The agreement of the volunteers at a location for built-up and non-built-up categories by number of times a location was skipped when a location was classified four times.

|  | 4 agreed | | 3 agreed | | 2 agreed | | |
| --- | --- | --- | --- | --- | --- | --- | --- |
|  | Built-up | Non-built-up | Built-up | Non-built-up | Built-up | Non-built-up | Equal Built-up/  Non-built-up |
| No skipping | 18 | 514 | 14 | 6 | N/A | N/A | 6 |
| 1 skipped | N/A | N/A | 2 | 81 | 3 | 1 | N/A |
| 2 skipped | N/A | N/A | N/A | N/A | 0 | 31 | 0 |

Skipped > 3 times = 3 locations; NA = not applicable

In addition, there were five locations in which only three answers were provided by the volunteers for the reasons outlined above. Of these five locations, three of them had 100% agreement that the location was non-built-up while two had 1 skipped answer and then 100% agreement that the location was non-built-up.

# ****Usage Notes****

The Database of Global Administrative Areas (GADM - https://gadm.org/) was used to calculate the number of points falling within each country and geographical sub-region.

Table S11: Number of points and density per country

| **Country** | **Number of points** | **Points per km^2^** |
| --- | --- | --- |
| Afghanistan | 768 | 0.0011897477 |
| Akrotiri and Dhekelia | 2 | 0.0085104570 |
| Åland | 9 | 0.0059951641 |
| Albania | 17 | 0.0005910638 |
| Algeria | 410 | 0.0001769789 |
| American Samoa | 1 | 0.0044498634 |
| Angola | 216 | 0.0001721088 |
| Anguilla | 2 | 0.0238791722 |
| Antarctica | 922 | 0.0000750362 |
| Argentina | 571 | 0.0002049136 |
| Armenia | 13 | 0.0004373703 |
| Australia | 1,123 | 0.0001454656 |
| Austria | 71 | 0.0008471694 |
| Azerbaijan | 30 | 0.0003474981 |
| Bahamas | 12 | 0.0008923393 |
| Bahrain | 2 | 0.0027851701 |
| Bangladesh | 92 | 0.0006570631 |
| Barbados | 1 | 0.0022869426 |
| Belarus | 60 | 0.0002903249 |
| Belgium | 35 | 0.0011412733 |
| Belize | 4 | 0.0001809112 |
| Benin | 167 | 0.0014377217 |
| Bhutan | 7 | 0.0001798852 |
| Bolivia | 173 | 0.0001587943 |
| Bonaire, Sint Eustatius and Saba | 6 | 0.0184242371 |
| Bosnia and Herzegovina | 24 | 0.0004698567 |
| Botswana | 83 | 0.0001429373 |
| Brazil | 1,757 | 0.0002054519 |
| Brunei | 2 | 0.0003438642 |
| Bulgaria | 37 | 0.0003313987 |
| Burkina Faso | 306 | 0.0011123551 |
| Burundi | 70 | 0.0025815493 |
| Cambodia | 245 | 0.0013427380 |
| Cameroon | 121 | 0.0002579363 |
| Canada | 1,859 | 0.0001872823 |
| Cape Verde | 4 | 0.0009722070 |
| Caspian Sea (with borders to several countries) | 78 | 0.0002097417 |
| Central African Republic | 382 | 0.0006119206 |
| Chad | 680 | 0.0005323896 |
| Chile | 191 | 0.0002516479 |
| China | 2,788 | 0.0002965500 |
| Colombia | 264 | 0.0002307087 |
| Comoros | 11 | 0.0065341231 |
| Cook Islands | 1 | 0.0038068409 |
| Costa Rica | 21 | 0.0004077591 |
| Côte d'Ivoire | 90 | 0.0002779949 |
| Croatia | 30 | 0.0005255873 |
| Cuba | 46 | 0.0004133940 |
| Cyprus | 2 | 0.0003488599 |
| Czech Republic | 51 | 0.0006476223 |
| Democratic Republic of the Congo | 2,314 | 0.0009869656 |
| Denmark | 32 | 0.0007435394 |
| Djibouti | 4 | 0.0001818298 |
| Dominica | 1 | 0.0013182577 |
| Dominican Republic | 20 | 0.0004136659 |
| Ecuador | 73 | 0.0002831224 |
| Egypt | 209 | 0.0002115946 |
| El Salvador | 17 | 0.0008274559 |
| Equatorial Guinea | 7 | 0.0002579152 |
| Eritrea | 120 | 0.0009904668 |
| Estonia | 16 | 0.0003528099 |
| Ethiopia | 1,024 | 0.0009008296 |
| Falkland Islands | 9 | 0.0007270906 |
| Faroe Islands | 2 | 0.0013944960 |
| Fiji | 4 | 0.0002097777 |
| Finland | 128 | 0.0003814701 |
| France | 390 | 0.0007099519 |
| French Guiana | 62 | 0.0007393557 |
| French Polynesia | 1 | 0.0002453097 |
| French Southern Territories | 8 | 0.0010177573 |
| Gabon | 42 | 0.0001576991 |
| Gambia | 36 | 0.0033536416 |
| Georgia | 20 | 0.0002861574 |
| Germany | 290 | 0.0008115475 |
| Ghana | 65 | 0.0002709846 |
| Greece | 79 | 0.0005950543 |
| Greenland | 521 | 0.0002435467 |
| Guadeloupe | 20 | 0.0120508788 |
| Guatemala | 42 | 0.0003825833 |
| Guernsey | 3 | 0.0339521286 |
| Guinea | 422 | 0.0017129613 |
| Guinea-Bissau | 64 | 0.0018773393 |
| Guyana | 24 | 0.0001136713 |
| Haiti | 108 | 0.0039631718 |
| Honduras | 40 | 0.0003541484 |
| Hong Kong | 3 | 0.0026439171 |
| Hungary | 41 | 0.0004408275 |
| Iceland | 21 | 0.0002063152 |
| India | 1,016 | 0.0003207617 |
| Indonesia | 535 | 0.0002811557 |
| Iran | 460 | 0.0002828402 |
| Iraq | 122 | 0.0002731935 |
| Ireland | 58 | 0.0008258015 |
| Israel | 19 | 0.0008548162 |
| Italy | 215 | 0.0007144732 |
| Jamaica | 11 | 0.0009945930 |
| Japan | 272 | 0.0007289704 |
| Jordan | 41 | 0.0004586422 |
| Kazakhstan | 517 | 0.0001894696 |
| Kenya | 100 | 0.0001695145 |
| Kosovo | 41 | 0.0037524482 |
| Kuwait | 20 | 0.0011461449 |
| Kyrgyzstan | 31 | 0.0001559231 |
| Laos | 47 | 0.0002032547 |
| Latvia | 23 | 0.0003563886 |
| Lebanon | 8 | 0.0007794157 |
| Lesotho | 10 | 0.0003262547 |
| Liberia | 187 | 0.0019368625 |
| Libya | 237 | 0.0001460763 |
| Lithuania | 29 | 0.0004470914 |
| Luxembourg | 2 | 0.0007755816 |
| Macedonia | 9 | 0.0003622430 |
| Madagascar | 507 | 0.0008527587 |
| Malawi | 276 | 0.0023244063 |
| Malaysia | 112 | 0.0003377757 |
| Mali | 709 | 0.0005635527 |
| Malta | 1 | 0.0030859529 |
| Martinique | 19 | 0.0168956464 |
| Mauritania | 157 | 0.0001500253 |
| Mauritius | 3 | 0.0014721542 |
| Mayotte | 11 | 0.0279155233 |
| Mexico | 647 | 0.0003299690 |
| Micronesia | 4 | 0.0051287642 |
| Moldova | 19 | 0.0005606241 |
| Mongolia | 231 | 0.0001475444 |
| Montenegro | 6 | 0.0004496942 |
| Montserrat | 2 | 0.0197606982 |
| Morocco | 111 | 0.0002676466 |
| Mozambique | 744 | 0.0009398235 |
| Myanmar | 134 | 0.0001992213 |
| Namibia | 146 | 0.0001763698 |
| Nepal | 212 | 0.0014303835 |
| Netherlands | 54 | 0.0014360595 |
| New Caledonia | 6 | 0.0003171180 |
| New Zealand | 82 | 0.0003049234 |
| Nicaragua | 37 | 0.0002868262 |
| Niger | 626 | 0.0005259664 |
| Nigeria | 287 | 0.0003139443 |
| North Korea | 343 | 0.0027909777 |
| Northern Cyprus | 23 | 0.0069247492 |
| Norway | 130 | 0.0004014762 |
| Oman | 137 | 0.0004406392 |
| Pakistan | 179 | 0.0002040740 |
| Palestina | 7 | 0.0011220682 |
| Panama | 18 | 0.0002381340 |
| Papua New Guinea | 102 | 0.0002185675 |
| Paracel Islands | 1 | 0.0505341436 |
| Paraguay | 74 | 0.0001843373 |
| Peru | 244 | 0.0001875480 |
| Philippines | 100 | 0.0003359402 |
| Poland | 199 | 0.0006380455 |
| Portugal | 51 | 0.0005543762 |
| Puerto Rico | 12 | 0.0013305082 |
| Qatar | 13 | 0.0011135095 |
| Republic of Congo | 77 | 0.0002238196 |
| Reunion | 20 | 0.0079201963 |
| Romania | 122 | 0.0005119683 |
| Russia | 2,913 | 0.0001726785 |
| Rwanda | 64 | 0.0025149720 |
| Saint Helena | 2 | 0.0048441631 |
| Saint Kitts and Nevis | 1 | 0.0037229527 |
| Saint Lucia | 1 | 0.0016178795 |
| Saint-Martin | 2 | 0.0354149032 |
| Samoa | 1 | 0.0003483811 |
| São Tomé and Príncipe | 1 | 0.0009910901 |
| Saudi Arabia | 821 | 0.0004265888 |
| Senegal | 49 | 0.0002474085 |
| Serbia | 30 | 0.0003834013 |
| Sierra Leone | 178 | 0.0024360669 |
| Slovakia | 27 | 0.0005503768 |
| Slovenia | 17 | 0.0008510255 |
| Solomon Islands | 12 | 0.0004178884 |
| Somalia | 772 | 0.0012105892 |
| South Africa | 431 | 0.0003521040 |
| South Georgia and the South Sandwich Islands | 5 | 0.0012231907 |
| South Korea | 53 | 0.0005277255 |
| South Sudan | 316 | 0.0004998521 |
| Spain | 229 | 0.0004520153 |
| Sri Lanka | 30 | 0.0004527257 |
| Sudan | 317 | 0.0001683698 |
| Suriname | 26 | 0.0001763884 |
| Svalbard and Jan Mayen | 4 | 0.0000666996 |
| Swaziland | 7 | 0.0004010634 |
| Sweden | 135 | 0.0003011067 |
| Switzerland | 35 | 0.0008489211 |
| Syria | 76 | 0.0004056600 |
| Taiwan | 30 | 0.0008202974 |
| Tajikistan | 27 | 0.0001897013 |
| Tanzania | 840 | 0.0008872571 |
| Thailand | 157 | 0.0003036574 |
| Timor-Leste | 6 | 0.0003996989 |
| Togo | 99 | 0.0017258782 |
| Trinidad and Tobago | 5 | 0.0009630942 |
| Tunisia | 50 | 0.0003220550 |
| Turkey | 275 | 0.0003517537 |
| Turkmenistan | 77 | 0.0001570582 |
| Uganda | 361 | 0.0014852117 |
| Ukraine | 305 | 0.0005076438 |
| United Arab Emirates | 62 | 0.0008667934 |
| United Kingdom | 193 | 0.0007878226 |
| United States | 3,618 | 0.0003817334 |
| United States Minor Outlying Islands | 1 | 0.0210415125 |
| Uruguay | 49 | 0.0002753412 |
| Uzbekistan | 82 | 0.0001830704 |
| Vanuatu | 3 | 0.0002421503 |
| Venezuela | 166 | 0.0001807430 |
| Vietnam | 123 | 0.0003714755 |
| Western Sahara | 110 | 0.0004096941 |
| Yemen | 97 | 0.0002132848 |
| Zambia | 157 | 0.0002079495 |
| Zimbabwe | 575 | 0.0014640245 |

Note that 4,843 points fell in the ocean so are not included in the above totals, but 96% of these points fall within 10 km^2^ of a coastline so could be allocated to countries.

Table S12: Number of points and density per geographical sub-region

| **Geographical sub-region** | **Number of points** | **Points per km^2^** |
| --- | --- | --- |
| Northern America | 5,999 | 0.00027845893 |
| Caribbean | 269 | 0.00114602376 |
| Central America | 826 | 0.00033276142 |
| South America | 3,683 | 0.00020641590 |
| Northern Europe | 783 | 0.00043351160 |
| Southern Europe | 749 | 0.00056854750 |
| Western Europe | 877 | 0.00079530202 |
| Eastern Europe | 3,774 | 0.00020297395 |
| Northern Africa | 1,444 | 0.00018880580 |
| Western Africa | 3,448 | 0.00056668144 |
| Eastern Africa | 5,975 | 0.00084849800 |
| Middle Africa | 3,840 | 0.00058105061 |
| Southern Africa | 677 | 0.00025255026 |
| Western Asia | 1,790 | 0.00039508078 |
| Central Asia | 734 | 0.00018313401 |
| Eastern Asia | 3,721 | 0.00032074055 |
| Southern Asia | 2,764 | 0.00041191160 |
| South-Eastern Asia | 1,461 | 0.00032552727 |
| Oceania | 1,340 | 0.00015677789 |
| Antarctica | 935 | 0.00007602029 |

Note that the 78 points that fall in the Caspian Sea have not been allocated to geographical sub-regions since this sea contains multiple national borders/sub-regions.
